# *Smi-miR164a* positively regulates phenolic acid biosynthesis while negatively regulates tanshinone production in *Salvia miltiorrhiza*

**DOI:** 10.3389/fpls.2026.1817574

**Published:** 2026-07-07

**Authors:** Huaqian You, Weibo Jin, Dongfeng Yang, Haihua Zhang, Zongsuo Liang

**Affiliations:** Key Laboratory of Plant Secondary Metabolism and Regulation of Zhejiang Province, College of Life Sciences and Medicine, Zhejiang Sci-Tech University, Hangzhou, China

**Keywords:** molecular breeding, phenolic acids, *Salvia miltiorrhiza*, Smi-miR164a, tanshinones, transcription regulation

## Abstract

**Introduction:**

MicroRNAs (miRNAs) are key post-transcriptional regulators of plant secondary metabolism. Their primary mechanism involves silencing target genes through mRNA cleavage or translational inhibition, which is a major focus of current research in this field. However, the specific regulatory roles of individual miRNAs in coordinating different secondary metabolic pathways in medicinal plants remain largely uncharacterized.

**Methods:**

This study investigated the roles of *Smi-miR164a* in *Salvia miltiorrhiza*. We generated *Smi-miR164a*-overexpressing (*OE-miR164a*) transgenic lines and performed comprehensive metabolic profiling and gene expression analysis.

**Results:**

Overexpression of Smi-miR164a resulted in significant accumulation of phenolic acids, with rosmarinic acid (RA) and salvianolic acid B (SalB) levels increased by up to 2.8-fold compared to wild-type (WT). Conversely, it markedly reduced the accumulation of tanshinones, decreasing tanshinone I (T-I) and tanshinone IIA (T-IIA) to 25-68% of WT levels. Transcriptional analysis showed that expression changes in key biosynthetic genes were tightly correlated with the metabolic alterations. Genes involved in the tanshinone pathway (e.g., *HMGR1, DXS2*) were downregulated, whereas those in the salvianolic acid pathway (e.g., *PAL1, C4H*) were upregulated, consistent with the reciprocal accumulation of their corresponding metabolites.

**Conclusion:**

These findings demonstrate that the *Smi-miR164a* module acts as a pivotal regulator, positively influencing phenolic acid biosynthesis while negatively regulating tanshinone production in *S. miltiorrhiza*. This gene presents a promising target for molecular breeding aimed at enhancing the yield of specific bioactive compounds.

## 1 Introduction

Tanshinones and salvianolic acids, as the important secondary metabolites of medicinal plant *Salvia miltiorrhiza* Bunge (Danshen, hereinafter referred to as *S. miltiorrhiza*), are the main medicinal components of *S. miltiorrhiza*-based drugs, which serve as the core therapeutic basis for clinical applications in cardiovascular and cerebrovascular diseases and diabetic nephropathy cardiovascular protection (Li et al., 2018; Ansari et al., 2021; Xu et al., 2023). But the yield of tanshinones and salvianolic acids is low, so the analysis of the metabolic synthesis pathway and transcription factor regulatory network is of great significance for elucidating the biosynthetic mechanism and improving the yield of tanshinones and salvianolic acids. A large number of omics analyses have basically annotated the key enzyme genes of metabolic pathways of tanshinones and salvianolic acids, and the studies on the regulation of tanshinones and salvianolic acids accumulation by transcription factor families of *S. miltiorrhiza MYB*, *bHLH*, and *WRKY* have been successively reported (Deng et al., 2023; Zhang et al., 2024a, 2017, 2024b).

Non-coding RNA (ncRNA) species, including circular RNAs (CircRNAs), long non-coding RNAs (lncRNAs), and microRNAs (miRNAs), have been shown to play pivotal regulatory roles in the biosynthesis of secondary metabolites in *S. miltiorrhiza* (Cai et al., 2019). MiRNA has been identified as a potential factor regulating gene expression. The identification of the substance in *S. miltiorrhiza*, along with its regulatory role in the secondary metabolites of the same organism, has been the subject of preliminary study (Shao and Lu, 2013; Zheng et al., 2020; Zou et al., 2021). It has been hypothesised that *SmMYBs* may be subject to regulation by miR159, miR319, miR828, and miR858. Furthermore, the subject was implicated in the biosynthesis of bioactive compounds of *S. miltiorrhiza* (Li and Lu, 2014). The MiR408-SmLAC3 module was found to participate in salvianolic acid B synthesis in *S. miltiorrhiza* (Zou et al., 2021). SmiR160a negatively regulates the biosynthesis of tanshinones in *S. miltiorrhiza* hairy roots by targeting Auxin Response Factors (*ARFs*) genes (Zhang et al., 2020).

With the discovery and identification of numerous medicinal plant miRNAs and growing research on their regulation of tanshinones and salvianolic acids, the biosynthesis of bioactive components in *S. miltiorrhiza* has emerged as a prominent research focus. Specifically, in model plants (e.g., *Arabidopsis*, rice), *miR164* targets NAC family genes (e.g., *CUC2*, *NAC60*) to regulate organ development and stress responses (Chen and Xia, 2025). In soybean, *miR164k* dynamically modulates salt tolerance by targeting *NAC1*—upregulating in sensitive cultivars to repress *NAC1*, while downregulating in salt-tolerant lines to activate *NAC1*—revealing conserved *miR164*-NAC crosstalk across species (Hernandez et al., 2020). In kiwifruit, ethylene suppresses *miR164* to relieve its inhibition of *NAC6/7*, thereby activating ethylene synthesis (*ACS1*/*ACO1*) and cell wall degradation (*MAN1*) genes during fruit ripening (Wang et al., 2020). However, despite these advances, the specific regulatory logic of the *miR164* in coordinating tanshinone and salvianolic acid biosynthesis remains uncharted in *S. miltiorrhiza*—prior studies on *miR164* in other species focused mainly on developmental roles.

Given the conserved role of *miR164* in regulating secondary metabolism across species (e.g., flavonoid synthesis in *Scutellaria baicalensis*, fruit ripening in kiwifruit), we hypothesized that a similar regulatory module may exist in *S. miltiorrhiza* to coordinate tanshinone and salvianolic acid biosynthesis. The Plant *miR164*-NAC module participates in multiple processes, including plant architecture in cotton (Zhan et al., 2021), disease defense in *Populus* (Chen et al., 2021), drought resistance in rice (Fang et al., 2014), and negatively regulates resistance to stripe rust in wheat (Feng et al., 2014). While *miR164*-NAC modules are conserved in plants, their function in coordinating tanshinone/salvianolic acid biosynthesis is entirely unreported in *S. miltiorrhiza*. This study bridges this gap by: 1) Validating the role of *miR164a* via transgenic assays; 2) Deciphering how this gene balances two metabolic pathways—a novel regulatory paradigm in medicinal plants. In this study, the *Smi-miR164a* gene obtained from *S. miltiorrhiza* is identified as a homologous of *Arabidopsis ath-miR164a* through a search of the miRBase (Release 22.1). Further analysis reveals a potential target gene.

The present study investigates the regulatory role of *miR164a* in the biosynthesis of tanshinones and salvianolic acids. The overexpression vector containing *At-miR164a* is constructe and introduce into *S. miltiorrhiza hairy* roots via genetic transformation. The accumulation levels of tanshinones and salvianolic acids in the transgenic hairy roots are subsequently measured. Additionally, the expression of key genes involved in their biosynthetic pathways was analyzed. This study provides the first functional and mechanistic insights into how *Smi-miR164a* coordinately regulates these two metabolic pathways.

## 2 Materials and methods

### 2.1 Bioinformatic screening and validation of *Smi-miR164a* and its target genes

To identify potential target genes of *Smi-miR164a*, we employed a multi-step pipeline combining small RNA-seq based miRNA identification, in silico target prediction, and degradome sequencing validation, as detailed below.

#### 2.1.1 Identification of *Smi-miR164a* from small RNA sequencing data

Conserved and novel miRNAs in *S. miltiorrhiza* were identified following the pipeline described by Shao et al. (2016). Briefly, raw small RNA sequencing reads were first processed by adapter trimming and quality filtering to obtain clean reads. Clean reads were then aligned against the Rfam database to remove non-miRNA sequences, including rRNA, snoRNA, snRNA, and tRNA. Remaining high-quality reads were mapped to the precursor database in miRBase 21.0; sequences with matches were annotated as known miRNAs. For reads that did not match known precursors, novel miRNAs were predicted based on characteristic miRNA secondary-structure features. Finally, unmapped sequences were aligned to the *S. miltiorrhiza* transcriptome (downloaded from http://bi.sky.zstu.edu.cn/Bio111/DsTRD/home.php) using Bowtie to further support the identification of miRNA precursors. Through this pipeline, *Smi-miR164a* was identified as a conserved miRNA with high expression levels in this study.

#### 2.1.2 Transcriptome data acquisition and miRNA target gene prediction

To predict potential targets of *Smi-miR164a*, coding sequences (CDS) of *S. miltiorrhiza* were first downloaded as the `codingRNA.fa` FASTA file from the *S. miltiorrhiza* transcriptome database DsTRD (http://bi.sky.zstu.edu.cn/DsTRD/home.php), which served as the reference library for target prediction. Subsequently, the plant miRNA target prediction tool psRNATarget (originally available at https://www.zhaolab.org/psRNATarget/, currently updated to https://www.zhaolab.org/psRNATarget/) was used for prediction.

Analysis was performed using the default V2 scoring system of psRNATarget, with key parameters set to balance sensitivity and specificity: Expectation ≤ 5, maximum UPE (unpaired energy) value ≤ 25, and a maximum of 2 mismatches allowed in the seed region (positions 2–13 nt of the mature miRNA sequence). Additional penalty parameters were configured as follows: 0.5 penalty for G:U pairs, 1.0 penalty for other mismatches, 2.0 penalty for gap opening, and 0.5 penalty for gap extension. The lengths of the upstream and downstream flanking sequences included in the analysis were set to 17 nt and 13 nt, respectively.

#### 2.1.3 Degradome sequencing data processing pipeline

To preliminarily validate the predicted target sites, previously published degradome sequencing data of *S. miltiorrhiza* were downloaded from the NCBI Sequence Read Archive (SRA) under accession number SRR1557864 (Xu et al., 2014). Raw reads were subjected to quality control: sequencing adapters and low-quality reads were removed, and clean reads were analyzed using the CleaveLand pipeline (Addo-Quaye et al., 2009) to characterize the 5’ end distribution of degradome fragments and identify *Smi-miR164a*-mediated specific cleavage sites. By comparing psRNATarget prediction results with significant cleavage signal peaks detected in the degradome data, potential target genes supported by experimental evidence were screened.

### 2.2 Culture and treatment of hairy roots

The Tasly R&D Institute (Tasly Holdings Group Co. Ltd., Tianjin, China) supplied the violet-flowered *S. miltiorrhiza* seeds for this research. These seedlings are obtained through subcultures of the preserved lines in our laboratory. Sterile seedlings that have been growing for one month are cut off from the internodes, and the stem segments with axillary buds are placed on 1/2 MS medium for subculture. The method of obtaining hairy roots is according to Pei et al (Pei et al., 2017). Culture was initiated by transferring 0.2 g of fresh hairy roots to a 150 mL flask with 50 mL of 6,7-V liquid medium. Incubation was performed for 28 days at 25 °C in the dark on a shaker operating at 110 rpm/min.

### 2.3 Plant expression vector construction

The Gateway-compatible entry vector *pDONR207*, destination vectors pK7WG2R (overexpression), along with the binary vector *pCAMBIA1300*, were maintained in our laboratory stock for genetic engineering. The mature *miR164a* sequence is identical in *S. miltiorrhiza* and *Arabidopsis thaliana* (Gao et al., 2025). Given the high conservation of the *miR164* family and its DCL1-mediated processing mechanism across angiosperms (Gao et al., 2025; Alvarez et al., 2006; Shao et al., 2015), the *Arabidopsis thaliana* pre*-miR164a* precursor was employed for heterologous overexpression. This strategy is predicated on the established principle that conserved miRNAs can be functionally reconstituted in related species using heterologous precursors, which are accurately processed to yield mature, biologically active miRNAs (Zhan et al., 2021).

Therefore, to overexpress *At-miR164a*, a corresponding recombinant plasmid was engineered. The upstream primer Pre-*miR164a*-F (containing Xba I restriction site) and the downstream primer Pre-*miR164a*-R (containing Kpn I restriction site) were designed based on the precursor sequence of *Arabidopsis* (Accession: MI0000197). The precursor sequence of *miR164a* was amplified to construct the overexpression vector pre-*MIR164a*-1300. The sequences of all primers used in this study are listed in **Supplementary Table 1**.

### 2.4 Hairy root transformation and transgenic line verification

The procedure for inducing transgenic hairy roots followed the method of Zhang et al. (2012), with some adaptations. The recombinant overexpression vectors pCAMBIA-1300-*miR164a* were introduced into *Agrobacterium tumefaciens*. Subsequently, sterile seedlings of *S. miltiorrhiza* were infected with the transformed bacteria to induce hairy root formation.

The presence of transgenic hairy roots was confirmed through polymerase chain reaction (PCR) analysis. Specific primers targeting the *rolB* and *rolC* genes from the *Agrobacterium* Ri plasmid, as well as the *NPT II* gene from the overexpression vector, were uesed for this verification. Rolb-F/Rolb-R (423 bp rolb fragment), Rolc-F/Rolc-R (626 bp rolc fragment), and NPT II-F/NPT II-R (497bp NPT II gene fragment from the overexpression vector). Additionally, positive plants exhibited successful amplification of the corresponding fragments, while in the wild-type plant lines, the *rol b* and *rol c* carried by the *Agrobacterium* Ri plasmid were successfully amplified, but the vector and target gene fragments were not detected.

### 2.5 The extraction and determination of salvianolic acids and tanshinones

The dried root (0.02 g) was ground into powder and immersed in 4 ml of 70% methanol in the dark overnight. Following the incubation period, the samples were subjected to sonication for 40 min. Thereafter, they were subjected to a centrifugation process at a speed of 8,000 * g for 10 min. The sample was then filtered through a 0.45 μm organic membrane filter, after which 10 μL of the filtrate was taken for HPLC analysis. HPLC conditions were based on the established laboratory method for the determination of tanshinones and salvianolic acid components in *S. miltiorrhiza* (Wang et al., 2014).

### 2.6 Quantification by qRT–PCR

RNA extraction was performed using the TIANGEN^®^ RNAprep Pure kit (Beijing Tiangen Biochemical Technology Co., Ltd.), which is designed for polysaccharide- and polyphenol-rich plant total RNA extraction, following the manufacturer’s instructions. First-strand cDNA synthesis was conducted employing the PrimeScript RT Reagent Kit (Takara Biomedical Technology, China). For miRNA reverse transcription, the poly-A-tailing method was employed. To remove potential genomic DNA contamination, 1 μg of total RNA was subjected to digestion using gDNA Eraser. Subsequently, PolyA polymerase (NEB, China) was used to add a Poly (A) tail to the 3’ end of miRNA, using *E. coli* Poly (A) polymerase and ATP. Reverse transcription was then performed using Oligo-dT with a universal sequence as the reverse transcription primer. Quantitative PCR was conducted using the SYBR^®^ Premix Ex Taq™ II RNaseH Plus kit from Dalian Bao Biological Products Co., Ltd. Takara employed a two-step PCR amplification method commencing at 95 °C for 45 seconds, followed by 40 cycles of 95 °C for 5 seconds and 59 °C for 34 seconds. *SmActin* and *SmU6* were utilised as reference genes for mRNA and microRNA real-time quantitative PCR, respectively. All transgenic hairy root samples were analyzed in triplicate, with each of the three independent biological replicates including three technical repeats. Quantification of relative gene expression was performed according to the 2^−ΔΔCt^ method, withing *SmActin* and *SmU6* used as internal control genes for normalization (Livak and Schmittgen, 2001).

### 2.7 Primers

All primer sequences used for vector construction and molecular verification in this study are provided in **Supplementary Table 1**. Include NPT II-specific primers: Used for identifying the overexpression vector backbone; JD35S-F: An upstream primer located within the CaMV 35S promoter, used for verifying recombinant vectors.

## 3 Results

### 3.1 Bioinformatic and functional preliminary analysis of *Smi-miR164a* in *S. miltiorrhiza*

**Figure 1:**
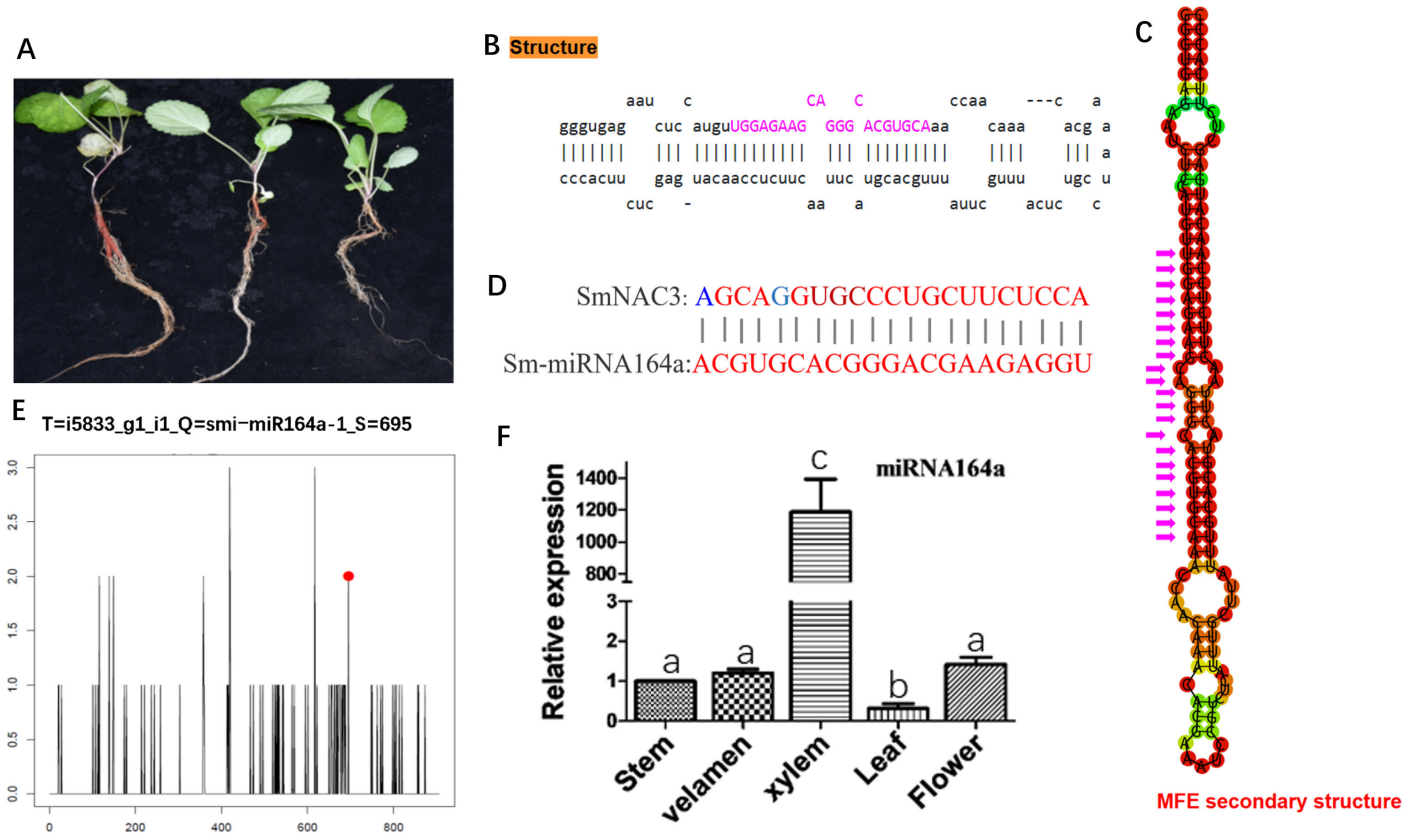
Bioinformatic and functional analysis of *Smi-miR164a* in *S. miltiorrhiza.* **(A)** Representative morphology of *S. miltiorrhiza* plants used in this study, showing the typical root and leaf structures of the experimental materials. **(B)** Sequence annotation of the pre-*miR164a* precursor, with the mature *Smi-miR164a* sequence highlighted in pink. **(C)** Minimum free energy (MFE) secondary structure prediction of the *Arabidopsis thaliana* pre-*miR164a* (*ath-MIR164a*, miRBase Accession No. MI0000197) precursor using RNAfold. The mature *Smi-miR164a* sequence is labeled with pink arrows, located in the double-stranded stem region, consistent with the canonical stem-loop structural characteristics of plant miRNA precursors. **(D)** Sequence alignment between *Smi-miR164a* and its potential target transcript *SmNAC3*, showing complementary base-pairing. **(E)** Degradome sequencing t-plot analysis of *SmNAC3*, a target of *Smi-miR164a*. The plot reveals a cleavage peak at the predicted target site, providing experimental evidence for the targeting regulatory relationship between *Smi-miR164a* and *SmNAC3*. **(F)** Tissue-specific expression pattern of *Smi-miR164a* in different tissues of *S. miltiorrhiza* (stems, periderm, xylem, leaves, and flowers). Data are presented as mean ± standard deviation (n=3). Different lowercase letters indicate significant differences between groups (p < 0.05).

Representative morphology of the *S. miltiorrhiza* plants used in this study is shown in **Figure 1A**, clearly presenting the typical root and leaf structures of the experimental materials. To verify the validity of the pre-*miR164a* precursor (*ath-MIR164a*, miRBase Accession No. MI0000197) used for genetic transformation, we predicted its secondary structure using the RNAfold web server (http://rna.tbi.univie.ac.at/cgi-bin/RNAWebSuite/RNAfold.cgi) (**Figures 1B, C**). **Figure 1B** shows the sequence annotation of the pre-*miR164a* precursor, where the mature *Smi-miR164a* sequence (21 nucleotides, consistent with the *S. miltiorrhiza* transcriptome assembly, identical to *Ath-miR164a*, miRBase Accession No. MIMAT0000164; obtained from miRBase: https://www.mirbase.org/) is highlighted in pink, clearly indicating its position within the precursor. **Figure 1C** presents the minimum free energy (MFE) stem-loop structure predicted by RNAfold, with the mature *Smi-miR164a* sequence labeled by pink arrows. The mature sequence is located in the double-stranded stem region of the precursor, consistent with the canonical structural characteristics of plant miRNA precursors, confirming that pre-*miR164a* can be correctly processed into mature miRNA by endogenous Dicer-like enzymes in plants.

With the validity of the pre-*miR164a* confirmed, we next performed target gene prediction for *Smi-miR164a* in *S. miltiorrhiza*. To predict the target genes of *Smi-miR164a* in *S. miltiorrhiza*, we used online analysis software (https://www.zhaolab.org/psRNATarget/), *S. miltiorrhiza* transcriptome data (http://bi.sky.zstu.edu.cn/DsTRD/home.php), and degradome sequencing data (accession number: SRR1557864). This analysis identified multiple potential targets related to transcriptional regulation and secondary metabolism, including NAC family transcription factors. The sequence alignment between *Smi-miR164a* and one of its potential target mRNAs, *SmNAC3* (**Figure 1D**) showed complementary base-pairing, supporting a potential targeting relationship. The degradome t-plot (**Figure 1E**) revealed a cleavage peak at the predicted site, providing in silico evidence supporting the targeting relationship. These complementary lines of evidence (sequence alignment and degradome analysis) provided a basis for subsequent functional investigation of *miR164a*.

Since the endogenous pre-*miR164a* sequence in *S. miltiorrhiza* was unknown, a 128-bp precursor sequence corresponding to *ath-MIR164a* (miRBase Accession No. MI0000197) was cloned from the *Arabidopsis* genome for genetic transformation. To determine the tissue-specific expression profile of *miR164a*, we performed qRT-PCR analysis. The mature miRNA abundance was quantified across five distinct tissues of *S. miltiorrhiza*: stems, leaves, flowers, periderm, and xylem. Although *miR164a* showed ubiquitous expression in the five tissues analyzed, its transcript levels displayed distinct expression profiles. Quantitative analysis revealed that the miRNA showed the lowest and highest accumulation in leaf and xylem, respectively (**Figure 1F**). While *miR164a* was highly expressed in xylem, tanshinones were mainly synthesized in periderm. This spatial dissociation between the miRNA expression site and the metabolite biosynthesis site suggests a potential complex, non-cell-autonomous regulatory mechanism.

It should be noted that this study only completed the bioinformatic prediction and preliminary degradome sequencing validation of *Smi-miR164a* target genes. Follow-up *in vitro* and *in vivo* functional validation experiments (e.g., dual-luciferase reporter gene assays, target gene overexpression/silencing analyses) are planned as priorities for future research.

### 3.2 Generation of transgenic hairy root lines with *miR164a*.

**Figure 2:**
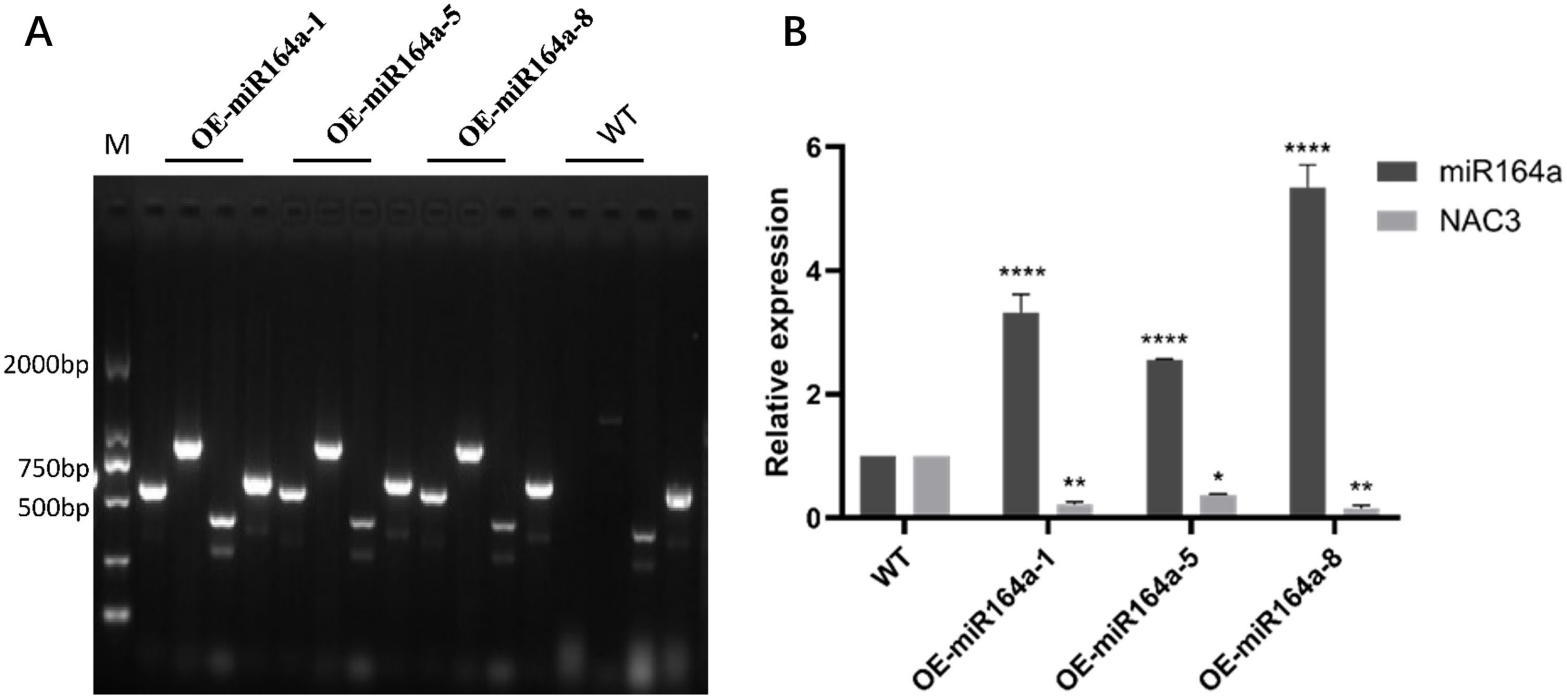
Molecular verification of *Smi-miR164a* overexpression in transgenic hairy root lines. **(A)** PCR verification of transgenic lines. M: DNA marker; WT: wild-type; OE-1, OE-5, OE-8: independent *Smi-miR164a*-overexpressing lines. **(B)** qRT-PCR analysis of *Smi-miR164a* and *SmNAC3* expression in *Smi-miR164a*-overexpressing hairy root lines. The relative expression of each gene in wild-type (WT) plants was normalized to 1. Values represent the mean ± standard deviation (SD) of three biological replicates. Asterisks denote significant differences vs. WT: *P < 0.05, **P < 0.01, ****P < 0.0001.

In order to study the regulatory effect of *miR164a* on the biosynthesis of phenolic acid and tanshinone in *S. miltiorrhiza*, transgenic hairy roots of *miR164a* were obtained through *Agrobacterium rhizogenes* ATCC15834-mediated leaf disc transformation. PCR and qRT-PCR were used to identify transgenic hairy root lines. The hair roots cultured ATCC15834 infection without plasmid were used as a control. The positive transgenic hairy roots were identified using gene-specific primers to amplify the CaMV 35S promoter and partial 164a gene; the wild-type hairy root lines only showed bands for the *rol b* and *rol c* genes (**Figure 2A**). The positive transgenic lines were subjected to PCR verification using a combination of four pairs of primers: rolB, rolC, hptII, and 35S+pre-*miR164a*. The rolB and rolC primers were sourced from the ATCC Ri plasmid, while the hptII primer was derived from the neomycin phosphotransferase II gene. The 35S+pre-*miR164a* primer was obtained from the 35S promoter and the 128 bp *ath-miR164a* sequence. The qRT-PCR results revealed significant differences in the expression levels of *miR164a* in the OE-*miR164a* lines compared to the WT, as illustrated in **Figure 2B**. Further detection showed that the expression level of NAC3 was significantly decreased in these overexpression lines, showing an obvious negative correlation with the expression change of *miR164a*, which further confirmed the targeted inhibitory relationship between *miR164a* and NAC3. Based on the expression levels of the target genes in transgenic hairy roots, three *miR164a* overexpression lines (OE-*miR164a*-1, OE-*miR164a*-5, and OE-*miR164a*-8) were used for the next study.

### 3.3 *Smi-miR164a* overexpression inhibits hairy root growth and biomass accumulation

**Figure 3:**
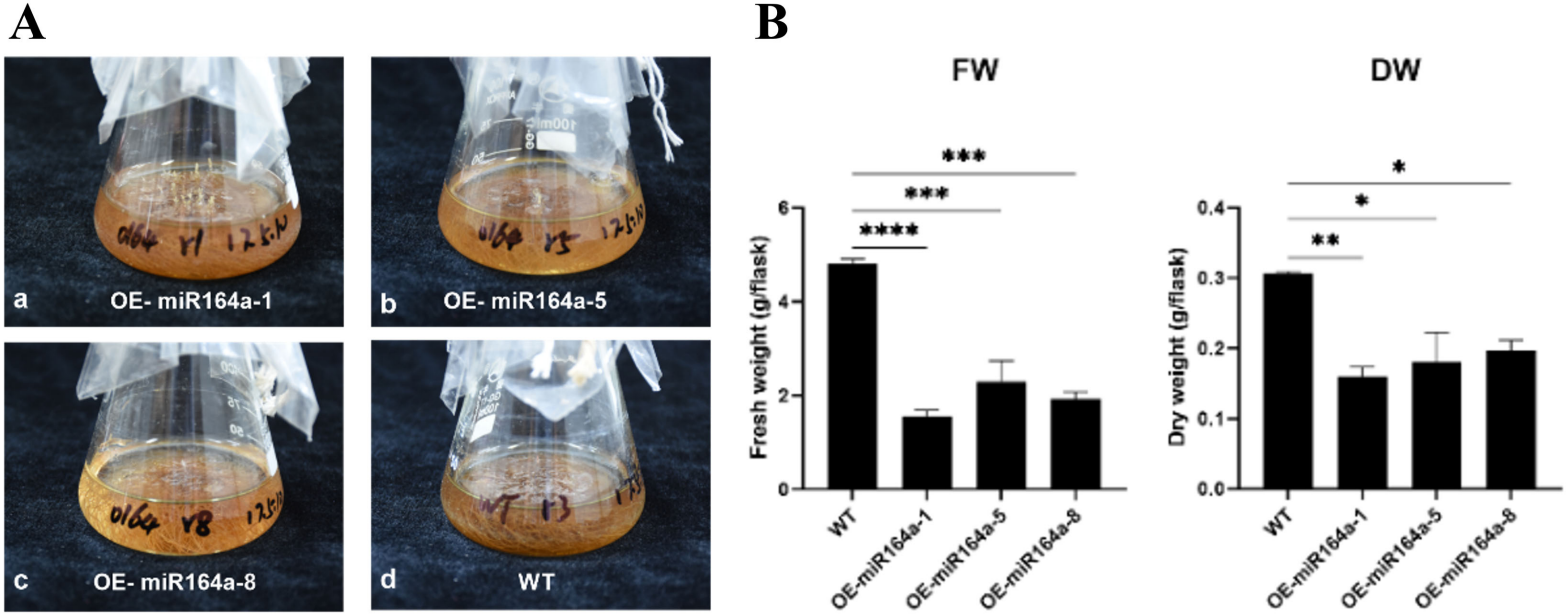
Growth phenotypes and biomass analysis in transgenic *S. miltiorrhiza* hairy roots. Quantitative comparison of fresh weight (FW) and dry weight (DW) (g/flask). *, **, and *** indicate statistically significant differences compared with WT at p < 0.05, p < 0.01, and p < 0.001, respectively. Data are presented as mean ± SD.

Genetic manipulation of *miR164a* induced divergent regulatory effects on hairy root biomass accumulation in *S. miltiorrhiza*. Genetic overexpression of *Smi-miR164a* profoundly inhibited the growth of *S. miltiorrhiza* hairy roots. As shown in **Figure 3A**, Smi*-miR164a*-overexpressing (OE) lines exhibited a severely stunted phenotype with sparse root systems and lighter-colored culture media, in stark contrast to the dense, dark-grown wild-type (WT) roots. This morphological suppression correlated with a significant reduction in biomass. Quantitative analysis confirmed that the fresh weight of OE lines was decreased by approximately 60% compared to the WT control (P < 0.001), while the dry weight was reduced to about 70% of the WT level (**Figure 3B**). These results demonstrate that *Smi-miR164a* acts as a strong negative regulator of hairy root growth and biomass accumulation.

### 3.4 *MiR164a* modulates phenolic acid and tanshinone biosynthesis in *S. miltiorrhiza*

**Figure 4:**
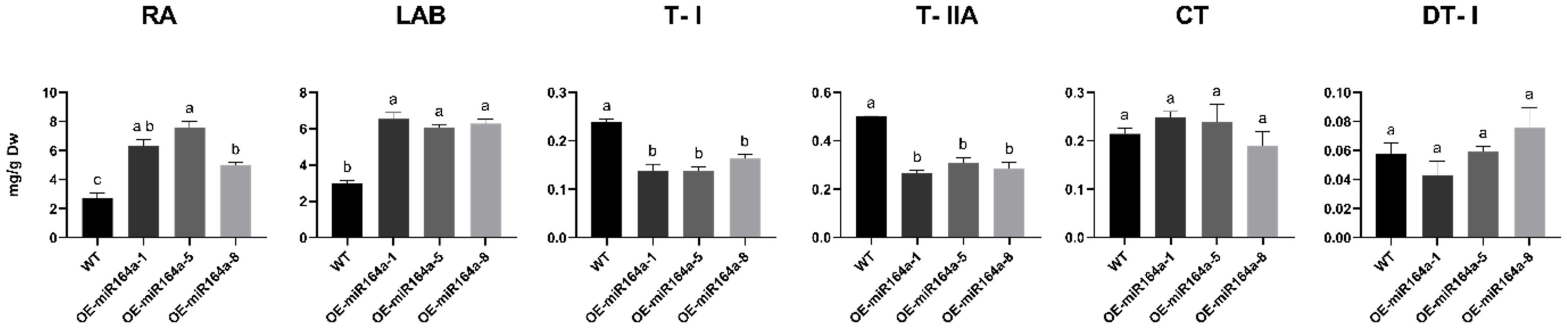
Differential accumulation of tanshinones and salvianolic acids in genetically modified *S. miltiorrhiza* hairy roots. Differences in the content of the tanshinone and salvianolic acids from OE-*miR164a*-1, 5, 8. All quantitative data are presented as mean ± standard deviation from three independent biological replicates. Different lowercase letters above the bars indicate statistically significant differences between groups (p < 0.05), as analyzed by one-way ANOVA followed by Tukey's multiple comparison test.

To investigate whether *miR164a* regulates the accumulation of tanshinones and salvianolic acids, the content of these compounds in hairy roots of *S. miltiorrhiza* was determined using HPLC after gene overexpression. The results showed that the content of SalB and RA was approximately 2.2- and 2.3-fold, 2.0- and 2.8-fold, and 2.1- and 1.8-fold higher in the OE-*miR164a*-1, 5, and 8 lines than in WT, respectively. In tanshinones, T-I and T-IIA decreased to 57.60% and 53.18%, 58.08% and 66.61%, 68.8% and 56.69% compared to the wild type. However, *miR164a* overexpression did not significantly alter the accumulation of CT or DT-I compared to WT (**Figure 4A**).

### 3.5 *MiR164a* regulates the expression of key biosynthetic genes in tanshinone and salvianolic acid pathways in *S. miltiorrhiza*

**Figure 5:**
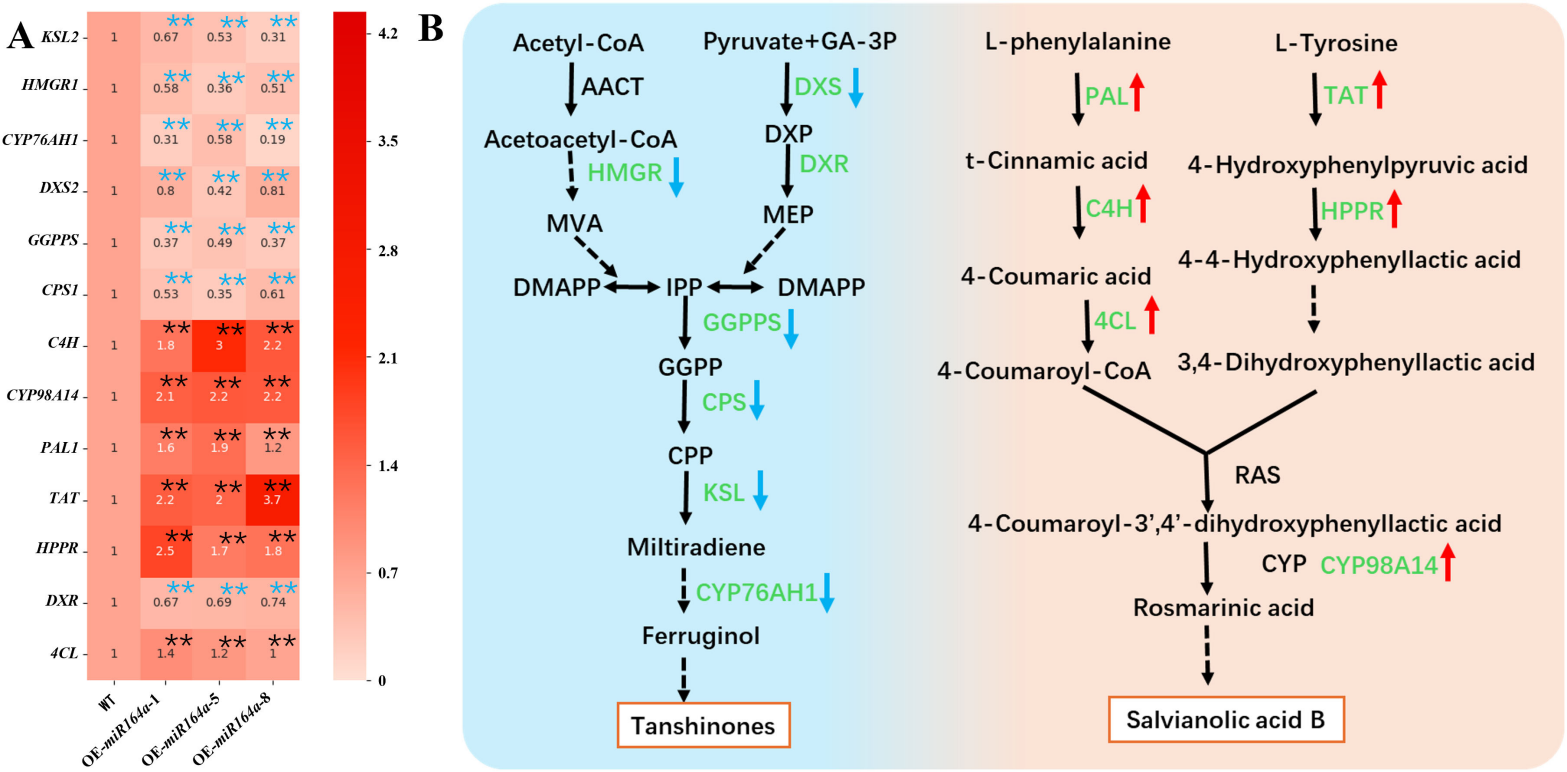
Differential expression of tanshinone and salvianolic acid B biosynthetic genes mediated by Smi-*miR164a* in transgenic *S. miltiorrhiza* hairy roots. **(A)** Expression heatmap of key biosynthetic genes. The gene expression data were quantified by quantitative real-time PCR (qRT-PCR). Relative transcript abundance in wild-type (WT) was normalized to 1. OE-miR164a-1, -5 and -8 denote three independent Smi-miR164a overexpression hairy root lines. Blue double asterisks (**) significantly downregulated at P < 0.01 vs WT; Black double asterisks (**) significantly upregulated at P < 0.01 vs WT. No markers are assigned to wild-type (WT) group. **(B)** Biosynthetic pathway diagrams of tanshinones and salvianolic acids. Red upward arrows (↑) indicate upregulated genes, and blue downward arrows (↓) indicate downregulated genes in *miR164a*-overexpressing lines (vs. WT). The tanshinone biosynthetic pathway in *S. miltiorrhiza* is based on previous studies (Xu et al., 2015; Guo et al., 2016). The salvianolic acid B biosynthesis pathway of phenolic acids in *S. miltiorrhiza* is based on previous studies (Xiao et al., 2009; Xu et al., 2016).

The biosynthetic pathways of tanshinones and salvianolic acids in *S. miltiorrhiza* have been well characterized in previous studies (tanshinones: Xu et al., 2015; Guo et al., 2016; salvianolic acid B: Xiao et al., 2009; Xu et al., 2016). To elucidate the regulatory role of *miR164a* in secondary metabolism, we quantified the relative transcript abundance of key enzyme genes involved in these two biosynthetic pathways via qRT-PCR. In the *miR164a*-overexpressing hairy root lines, the expression levels of key enzyme genes (*HMGR1, DXS2, DXR, GGPPS1, CPS1, KSL2*, and *CYP76H1*) in the tanshinone biosynthetic pathway were downregulated, while the expression levels of key enzyme genes (*PAL1, C4H, 4CL*, *CYP98A14, TAT*, and *HPPR*) in the salvianolic acid biosynthetic pathway were upregulated (**Figure 5**).

## 4 Discussion

Previous studies on *S. miltiorrhiza* secondary metabolism have primarily focused on structural genes involved in core biosynthetic pathways, while upstream regulatory networks, especially the roles of miRNAs in coordinating the balance between different metabolite branches, remain poorly understood. 

Zhu et al. (2024) identified that genes from the UBC family in Salvia castanea exert reciprocal regulation on the biosynthesis and accumulation of phenolic acids and tanshinones in Salvia miltiorrhiza, supporting the existence of regulatory trade-offs between distinct secondary metabolic branches. In line with this antagonistic regulatory mode, Zhang et al. (2024) further validated that ScWRKY35, a transcription factor isolated from S. castanea, oppositely modulates the production of phenolic acids and tanshinones while participating in abiotic stress adaptation in transgenic S. miltiorrhiza hairy roots. Additionally, SmWRKY33 was proven to coordinate tanshinone biosynthesis by modulating precursor flux and metabolic branch point activity in S. miltiorrhiza (Li et al., 2026). Beyond Salvia species, a comparable metabolic trade-off within flavonoid pathways has been documented in cotton: loss-of-function mutation of GhOMT1 drives excessive anthocyanidin accumulation and diversifies fiber pigmentation, revealing conserved competitive relationships among flavonoid sub-branches (Ke et al., 2022). At the interspecific level, pronounced disparities in bioactive constituent accumulation between S. miltiorrhiza and its congener S. castanea are closely shaped by the assembly and dynamics of rhizosphere microbial communities (Xu et al., 2023). Phylogenetic divergence across the genus Salvia is also accompanied by clear differentiation in volatile secondary metabolites (Wang et al., 2024). Consistently, metabolomic profiling further uncovered substantial interspecific divergence in phenolic acid and volatile constituents between two representative Salvia species at the leaf tissue level (Wang et al., 2025). Collectively, these studies illustrate that competitive metabolic trade-offs are prevalent in the secondary metabolism of Salvia plants, which are governed by diverse transcription factors at the intraspecific level, while interspecific metabolic differentiation is jointly driven by phylogenetic evolution, rhizosphere microbiota and environmental adaptation.

This study identified *miR164a* as a key regulator that reciprocally modulates the biosynthesis of tanshinones and salvianolic acids, filling an important gap in miRNA-mediated secondary metabolic regulation in medicinal plants. The study reveals *Smi-miR164a* as a key metabolic switch that reciprocally regulates the biosynthesis of tanshinones (diterpenoids) and salvianolic acids (phenolics) in *S. miltiorrhiza*. The *miR164a*-mediated regulatory dichotomy provides a possibility of a novel mechanism for resolving metabolic trade-offs between these two major pathways through targeted transcriptional control. Key evidence includes: *miR164a* overexpression suppresses tanshinones while promoting salvianolic acids, correlating with the reciprocal expression of key pathway genes (**Figure 5**). The *miR164a*-mediated mechanism demonstrates a novel regulatory dichotomy in plant diterpenoid vs. phenolic biosynthesis, resolving metabolic trade-offs through targeted transcriptional control.

This study establishes a primary research direction focused on elucidating the role of *Smi-miR164a* as a master regulator in *S. miltiorrhiza*. It specifically investigates how *Smi-miR164a* orchestrates a reciprocal balance between the biosynthesis of diterpenoids (tanshinones) and phenolics (salvianolic acids). A central objective is to elucidate the molecular trade-offs governing this metabolic dichotomy. Given that miRNAs are known regulators of specialized metabolism in *S. miltiorrhiza* (Zhang et al., 2020), this study focuses on *Smi-miR164a*. The second major direction involves deciphering the mechanism by which *Smi-miR164a* reprioritizes resource allocation, leading to a shift from primary growth and biomass accumulation towards the enhanced production of specific secondary metabolites. Molecular evidence supporting this shift was obtained, wherein bioinformatic analysis identified *SmNAC3* as a putative target of *Smi-miR164a*. Specifically, we identified *SmNAC3* as a putative target gene of *Smi-miR164a* through a combination of in silico prediction using the psRNATarget online tool and preliminary validation with published degradome sequencing data of *S. miltiorrhiza*. A canonical miRNA-target regulatory relationship was observed between *Smi-miR164a* and *SmNAC3*. Concurrently, numerous studies in model plants such as *Arabidopsis thaliana* have demonstrated that *miR164* family members regulate plant growth and development by targeting NAC transcription factors, and this regulatory module is highly conserved across the plant kingdom. For instance, *miR164* in *Arabidopsis* has been shown to target multiple NAC family members, including CUC1/AtNAC054, *CUC2*/AtNAC098, *NAC1*/AtNAC021, ORESARA1 (ORE1)/NAC2/AtNAC092, NAC4/AtNAC079/080, and NAC5/At*NAC1*00 (Patrick et al., 2004; Mallory et al., 2004; Guo et al., 2005; Kaneda et al., 2009; Jones-Rhoades et al., 2006). These findings provide strong biological rationale supporting our predicted target interaction in *S. miltiorrhiza*, and thus prompted us to use the conserved *Arabidopsis* precursor of *miR164a* for functional investigation. Subsequently, transgenic hairy root lines overexpressing *Smi-miR164a* were successfully established and validated. Phenotypically, *Smi-miR164a* overexpression strongly inhibited hairy root growth, reducing fresh biomass by approximately 60%. At the metabolic level, this genetic perturbation reciprocally altered secondary metabolite profiles, enhancing phenolic acids (e.g., Salvianolic acid B and Rosmarinic acid) by approximately 2-fold while suppressing specific diterpenoids (e.g., Tanshinone I and IIA) to 50-70% of wild-type levels. Notably, we also quantified the contents of other major tanshinone components, including cryptotanshinone (CT) and dihydrotanshinone (DT), and no significant differences were observed between the *miR164a*-overexpressing hairy root lines and wild-type (WT) controls. Given the lack of significant changes, these data were not included in the main figures. This differential response indicates that *Smi-miR164a* exerts a branch-specific regulatory effect on secondary metabolism, rather than acting as a global regulator. Based on the metabolite accumulation patterns, we speculate that under the experimental conditions used here, the biosynthetic fluxes corresponding to CT and DT are likely insensitive to *Smi-miR164a*-mediated regulation, resulting in stable metabolic flow through these branches. Additionally, the activation of the phenolic acid biosynthetic pathway likely diverts a portion of the upstream carbon flux, which may further exacerbate the differences in accumulation patterns among different tanshinone components. This also reflects the inherent stability of the CT and DT biosynthetic branches in *S. miltiorrhiza*. Collectively, these results suggest that *Smi-miR164a* functions not as a global regulator, but rather as a branch-specific regulatory switch, highlighting the complexity and specificity of plant secondary metabolic networks.

The overexpression of *Smi-miR164a* simultaneously suppresses tanshinone pathway genes (e.g., HMGR1, DXS2, CPS1, KSL2) and activates salvianolic acid pathway genes (e.g., PAL1, 4CL, TAT). This pursuit aligns with established findings that miRNAs can orchestrate such shifts by modulating target gene networks in medicinal plants (Jafari et al., 2023; Niu et al., 2024; Cao et al., 2024) or by influencing the development of metabolite-storing structures (Cao et al., 2024). We hypothesize that *Smi-miR164a* acts as a master switch by downregulating an upstream transcriptional regulator. This putative regulator likely promotes tanshinone biosynthesis while repressing the salvianolic acid pathway. Thus, *Smi-miR164a*-mediated inhibition of this factor coordinately redirects metabolic flux, explaining the observed reciprocal gene expression pattern. This study reveals for the first time that *Smi-miR164a* acts as a core metabolic switch, capable of reversibly regulating the biosynthesis of two important medicinal components in *S. miltiorrhiza*—tanshinones (diterpenoids) and salvianolic acids (phenolic acids)—thereby offering a novel framework for understanding how plants coordinate the synthesis of different defense metabolites (Li et al., 2018; Zhao et al., 2004). Traditional research has predominantly focused on the direct regulation by transcription factors (such as MYB, bHLH) (Chen et al., 2020). In contrast, this study found that *Smi-miR164a* selectively inhibits key genes in the tanshinone pathway (such as CPS1, KSL2) while activating genes in the salvianolic acid pathway (such as PAL1, 4CL), revealing a more refined, microRNA-mediated post-transcriptional regulatory hierarchy. This “see-saw” pattern indicates that plants can reprogram metabolic flux by regulating a single miRNA node, providing a new molecular model for unraveling the fundamental scientific question of secondary metabolic balance (Xing et al., 2025; Liu et al., 2025).

At the application level, this discovery provides a key target for the precise improvement of *S. miltiorrhiza* herb quality through genetic engineering. Compared to traditional breeding or chemical induction methods, genetic manipulation targeting *Smi-miR164a* (such as regulating its expression level) enables directed reprogramming of metabolic flux, thereby allowing for the optimization of the ratio and total content of tanshinones and salvianolic acids according to practical needs (Zheng et al., 2021). This offers a new strategy for cultivating high-medicinal-value *S. miltiorrhiza* varieties and enhancing herb quality, while also providing a valuable reference for the metabolic engineering improvement of other medicinal plants through the regulation of key miRNAs (Zheng et al., 2021). This study first demonstrates that *Smi-miR164a* acts as a core metabolic switch, reciprocally regulating the biosynthetic balance between tanshinones and salvianolic acids, thereby deepening the understanding of the secondary metabolic regulatory network in medicinal plants. However, the direct regulatory relationship between *Smi-miR164a* and its potential targets (e.g., *SmNAC3*) requires further experimental validation. Additionally, several key downstream genes involved in tanshinone biosynthesis, including *CYP76AH3*, *CYP76AK1*, the *CYP71D* family, and *TIIAS*, were not included in the scope of the current study. The primary focus of this work was on the upstream key nodes of the biosynthetic pathways that are directly regulated by *miR164a*, and the regulatory effects of *miR164a* on these downstream genes remain to be further explored in follow-up research. It should be noted that the core objective of this study was to investigate the regulatory effect of *Smi-miR164a* on the biosynthesis of phenolic acids and tanshinones in *S. miltiorrhiza*. Therefore, *in vitro* and *in vivo* functional validation of the regulatory relationship between *Smi-miR164a* and *SmNAC3* was not performed in this work. The in-depth dissection of this regulatory mechanism, including experimental validation of the miRNA-target interaction via double-overexpression assays, will be undertaken in future studies. Future research should involve functional complementation assays using miRNA-resistant versions of the target genes, combined with multi-omics approaches to systematically dissect its downstream regulatory network, providing novel targets for molecular design breeding of *S. miltiorrhiza* varieties (Li et al., 2018; Zheng et al., 2021).

## 5 Conclusion

This study elucidates the role of *miR164a* in regulating the biosynthesis of tanshinones and salvianolic acids, two critical secondary metabolites in *S. miltiorrhiza*. Our findings reveal that *miR164a* positively regulates salvianolic acid and rosmarinic acid biosynthesis but negatively impacts tanshinone accumulation. The modulation of key biosynthetic enzyme gene expression further corroborates the regulatory dynamics of this gene. These insights not only advance our understanding of the molecular mechanisms underlying secondary metabolite biosynthesis but also provide a potential strategy for enhancing the yield of specific bioactive compounds through genetic engineering. This work contributes to the broader application of miRNA-based regulation in improving the medicinal quality of plants. Future research could explore additional miRNA-transcription factor modules to optimize the metabolic pathways of medicinal plants for pharmaceutical use.

## Data Availability

The data supporting the findings of this study are available within the article and its **Supplementary Material**.

## Supplementary material

Supplementary Table 1 Directionally annotated primer sequences used in this study.
