# Supplementary material for: Smi-miR164a positively regulates phenolic acid biosynthesis while negatively regulates tanshinone production in Salvia miltiorrhiza
Source: Front Plant Sci. 2026 Jul 7;17:1817574. doi: 10.3389/fpls.2026.1817574 (PMC13385507; doi:10.3389/fpls.2026.1817574)
Supplement: Supplementary Table 1 — Directionally annotated primer sequences used in this study. [file DataSheet1.pdf]

>SRR1557864.1 HWI-D00318:201:H7P3BADXX:1:1101:1233:2089 length=33
GAGGAGAAGAAGCACGAAGGATTCATCGAGAAG
>SRR1557864.2 HWI-D00318:201:H7P3BADXX:1:1101:1500:2068 length=33
AAAGCCCAACTTGAGAATCGGGCGGCCACGCCG
>SRR1557864.3 HWI-D00318:201:H7P3BADXX:1:1101:1423:2111 length=33
TCTTTACCGTGTGGGATGTTGGTGGGCAAGAGA
>SRR1557864.4 HWI-D00318:201:H7P3BADXX:1:1101:1384:2131 length=33
CAAGCTGCTGCTGGACAATAGGTTGGGGAAGCA
>SRR1557864.5 HWI-D00318:201:H7P3BADXX:1:1101:1323:2211 length=33
AATTCATGGGGAGCTGACTGGGGGGACAATGGC
>SRR1557864.6 HWI-D00318:201:H7P3BADXX:1:1101:1723:2049 length=33
GAATGGGCTGAGATACGGCACCAGCTCTTCAAC
>SRR1557864.7 HWI-D00318:201:H7P3BADXX:1:1101:1520:2061 length=33
TCAAACGAGGAAAGGCTTACGGTGGATACCTAG
>SRR1557864.8 HWI-D00318:201:H7P3BADXX:1:1101:1512:2104 length=33
TTATGTCCTTTCATGAATGTGGAGGTAATGTTG
>SRR1557864.9 HWI-D00318:201:H7P3BADXX:1:1101:1587:2113 length=33
GAATTTCTGGTGGGCAGAAAAAGCGTGTCACAA
>SRR1557864.10 HWI-D00318:201:H7P3BADXX:1:1101:1608:2175 length=33
CATGCGTAAGCTCTTTCCAAGGTTCAAGGCTGA
>SRR1557864.11 HWI-D00318:201:H7P3BADXX:1:1101:1768:2055 length=33
GGTTCAACAACAGATCTCCCGGATACTACGATG
>SRR1557864.12 HWI-D00318:201:H7P3BADXX:1:1101:1788:2063 length=33
AGTTTTGCACGACGGGTTCCCCTGCCTCGAGGC
>SRR1557864.13 HWI-D00318:201:H7P3BADXX:1:1101:1891:2128 length=33
CGTCTTGATGTTGACTGCATTGATCTCTATTAT
>SRR1557864.14 HWI-D00318:201:H7P3BADXX:1:1101:1770:2136 length=33
CTCGGGCGACCGGAATGACGGGGCCGAACTAGG
>SRR1557864.15 HWI-D00318:201:H7P3BADXX:1:1101:1884:2159 length=33
ACATGCTTCCGATGTCAAGTGGAAATTCCTACT
>SRR1557864.16 HWI-D00318:201:H7P3BADXX:1:1101:1763:2199 length=33
AGGCTGAAACAAGGGGATAAGATGGCGAGCATT
>SRR1557864.17 HWI-D00318:201:H7P3BADXX:1:1101:1820:2219 length=33
CGCTGCACTCATCACTTCAAAGGATTGTGTAAA
>SRR1557864.18 HWI-D00318:201:H7P3BADXX:1:1101:2233:2069 length=33
TTGGATGTGGATGCTCTCTTCATTTCTCACATT
>SRR1557864.19 HWI-D00318:201:H7P3BADXX:1:1101:2062:2089 length=33
TCAAACGAGGAAAGGCTTACGGTGGATACCTAG
>SRR1557864.20 HWI-D00318:201:H7P3BADXX:1:1101:2085:2132 length=33
ACGGAAGGTACTGGACAATGTGGAAGCTGCCCA
>SRR1557864.21 HWI-D00318:201:H7P3BADXX:1:1101:2033:2149 length=33
GAACCACCGGGGCGGTCGGCACTCACGGTGCTG
>SRR1557864.22 HWI-D00318:201:H7P3BADXX:1:1101:2191:2215 length=33
TACGGCGAAATCGGAATCCCGGCATGAAATCTT

>SRR1557864.23 HWI-D00318:201:H7P3BADXX:1:1101:2088:2239 length=33
AGCTATGAGTACGATTTGAGCTACTTTGTTGCT
>SRR1557864.24 HWI-D00318:201:H7P3BADXX:1:1101:2490:2066 length=33
TCAAACGAGGAAAGGCTTACGGTGGATACCTAG
>SRR1557864.25 HWI-D00318:201:H7P3BADXX:1:1101:2302:2070 length=33
ACTTCTTGAATCTTTCAGGTGTTTACTAGAAGA
>SRR1557864.26 HWI-D00318:201:H7P3BADXX:1:1101:2465:2076 length=33
GACAGCTGGTAAAAGATACTTCAGCTAAACTTA
>SRR1557864.27 HWI-D00318:201:H7P3BADXX:1:1101:2252:2139 length=33
TACACTCCCTTCGTTCTACGAACCCTTGTTGAT
>SRR1557864.28 HWI-D00318:201:H7P3BADXX:1:1101:2470:2145 length=33
TCAAACGAGGAAAGGCTTACGGTGGATACCTAG
>SRR1557864.29 HWI-D00318:201:H7P3BADXX:1:1101:2338:2146 length=33
AGAATGCATTGGATGGATGCCCGGGCATTGAGA
>SRR1557864.30 HWI-D00318:201:H7P3BADXX:1:1101:2354:2223 length=33
TCAAACGAGGAAAGGCTTACGGTGGATACCTAG
>SRR1557864.31 HWI-D00318:201:H7P3BADXX:1:1101:2699:2074 length=33
ACAATACTGCGATTGGAAGGCTCGACAAGATGT
>SRR1557864.32 HWI-D00318:201:H7P3BADXX:1:1101:2610:2124 length=33
ACTTGAGTACCGTTCTTCCGCAGGTGGTTAAAT
>SRR1557864.33 HWI-D00318:201:H7P3BADXX:1:1101:2570:2136 length=33
GAAGCATTGTCTACTCTTAAAGAGCCAAATGGA
>SRR1557864.34 HWI-D00318:201:H7P3BADXX:1:1101:2567:2221 length=33
CACGGATCTGAATATCACCACAAGCAGCCGGCA
>SRR1557864.35 HWI-D00318:201:H7P3BADXX:1:1101:2766:2064 length=33
ATGTCGCTCGCCTCCTTCTACAACCCCGGCAGC
>SRR1557864.36 HWI-D00318:201:H7P3BADXX:1:1101:2755:2148 length=33
AGAATGCATTGGATGGATGCCCGGGCATTGAGA
>SRR1557864.37 HWI-D00318:201:H7P3BADXX:1:1101:2857:2202 length=33
ATTCGATCCCTACGCCTCCGACATCAACTATCT
>SRR1557864.38 HWI-D00318:201:H7P3BADXX:1:1101:2777:2216 length=33
CGTGCCATTTGGCGAAGCAAGCTTTTGATGAGG
>SRR1557864.39 HWI-D00318:201:H7P3BADXX:1:1101:3089:2059 length=33
TTGGAGGTGGCGGAGCACGCGGTGGTTTGGTGG
>SRR1557864.40 HWI-D00318:201:H7P3BADXX:1:1101:3056:2074 length=33
TCAAGGAGCTTCTCCTCAAATCCGGGTTCGAGA
>SRR1557864.41 HWI-D00318:201:H7P3BADXX:1:1101:3146:2090 length=33
GTTGTGGCAACTCCGATGAAAGTAACTTCCTTC
>SRR1557864.42 HWI-D00318:201:H7P3BADXX:1:1101:3035:2108 length=33
CCCCTACGTGTACAAATCACCGCCGCCGCCGAC
>SRR1557864.43 HWI-D00318:201:H7P3BADXX:1:1101:3191:2110 length=33
GTTTGGATCCTCTGCAACGACTGTGGAGAGACA
>SRR1557864.44 HWI-D00318:201:H7P3BADXX:1:1101:3238:2188 length=33
TAGATATGGAGATATGGATTCTGGTTACCGCCC

>SRR1557864.45 HWI-D00318:201:H7P3BADXX:1:1101:3163:2196 length=33
GTGAAGGTGAAAGTTGGGATGGAGATTTTGAGG
>SRR1557864.46 HWI-D00318:201:H7P3BADXX:1:1101:3244:2234 length=33
TATGGGGGAGGAGAAACGTTTGGTGGCATTCGA
>SRR1557864.47 HWI-D00318:201:H7P3BADXX:1:1101:3010:2246 length=33
GGAGCTCTTCTGCTTCAGATCGCGCGACCTCGT
>SRR1557864.48 HWI-D00318:201:H7P3BADXX:1:1101:3326:2052 length=33
AAAGGGAATCGGGTTAAAATTCCTGAACCGGGA
>SRR1557864.49 HWI-D00318:201:H7P3BADXX:1:1101:3429:2075 length=33
CATTGGTACAAAGTTGTTGAATCATATTCTTGA
>SRR1557864.50 HWI-D00318:201:H7P3BADXX:1:1101:3331:2113 length=33
AGAATGAAAGCCATTGTTGAAGCATCAGAAGGT
>SRR1557864.51 HWI-D00318:201:H7P3BADXX:1:1101:3476:2117 length=33
CAAGAAACACAAGAAGGATAAAGAGGAGGAAGA
>SRR1557864.52 HWI-D00318:201:H7P3BADXX:1:1101:3459:2122 length=33
ATCAGGAAACAACAGCTAGTGGCAAGTCTGGTT
>SRR1557864.53 HWI-D00318:201:H7P3BADXX:1:1101:3293:2142 length=33
AATTGGAGATAGCGGCGGCAAAAAAGGAAGGAA
>SRR1557864.54 HWI-D00318:201:H7P3BADXX:1:1101:3394:2212 length=33
CTCAACAGAAGAAGCTTAACCTTCCAATCCTCC
>SRR1557864.55 HWI-D00318:201:H7P3BADXX:1:1101:3355:2220 length=33
GTGGCATCTCGTAAGCACTACACAGTTGATGTT
>SRR1557864.56 HWI-D00318:201:H7P3BADXX:1:1101:3703:2037 length=33
TCGAAGGAGACACCTTCTGTGGTGCCCGATCCA
>SRR1557864.57 HWI-D00318:201:H7P3BADXX:1:1101:3733:2110 length=33
ATCTGCGGTCGGCGTTCGACGTTCTCGACGCCG
>SRR1557864.58 HWI-D00318:201:H7P3BADXX:1:1101:3574:2132 length=33
AACTTACAAGGATTCCCCTAGTAACGGCGAGCG
>SRR1557864.59 HWI-D00318:201:H7P3BADXX:1:1101:3550:2220 length=33
CGATGAGCTTCTCTCTAACTCATTCCCATAGGA
>SRR1557864.60 HWI-D00318:201:H7P3BADXX:1:1101:3958:2041 length=33
TCCGACGGCGCCGACGCTTCGCAGCTGAAATCA
>SRR1557864.61 HWI-D00318:201:H7P3BADXX:1:1101:3935:2069 length=33
GATCAAGAGAGTGACTCGCCGGAGAGTAATGAG
>SRR1557864.62 HWI-D00318:201:H7P3BADXX:1:1101:3771:2102 length=33
TCCTTCTCTAAAGAGGGGGCTGAAGAGGTGGAA
>SRR1557864.63 HWI-D00318:201:H7P3BADXX:1:1101:3911:2113 length=33
GGTACACGGAGCTGATGAACGGGATCATCGACA
>SRR1557864.64 HWI-D00318:201:H7P3BADXX:1:1101:3824:2156 length=33
TTGGAGTGCTCGAAAAGAGGGTGTTAATGGCAG
>SRR1557864.65 HWI-D00318:201:H7P3BADXX:1:1101:4105:2191 length=33
CAAGTATTACGAATACATGACTCCTTATGTCTC
>SRR1557864.66 HWI-D00318:201:H7P3BADXX:1:1101:4063:2243 length=33
GCAAAGGGCAAACAACGATAGCTAAAGAGTACT

>SRR1557864.67 HWI-D00318:201:H7P3BADXX:1:1101:4342:2039 length=33
AGTTCAAACAGCCGTGGTGAAGAGCAACTTGAA
>SRR1557864.68 HWI-D00318:201:H7P3BADXX:1:1101:4351:2053 length=33
AAGAATGCATTGGATGGATGCCCGGGCATTGAG
>SRR1557864.69 HWI-D00318:201:H7P3BADXX:1:1101:4463:2142 length=33
GAAGTGCATTCAGAACTTGCAGAATTTCAGAGG
>SRR1557864.70 HWI-D00318:201:H7P3BADXX:1:1101:4316:2232 length=33
TTCACGGCGAGTGTATAGAGAAGTGGCTGAATA
>SRR1557864.71 HWI-D00318:201:H7P3BADXX:1:1101:4739:2072 length=33
CGTGTTCGAGCCTAGTCTTGCCACCTATGAGAG
>SRR1557864.72 HWI-D00318:201:H7P3BADXX:1:1101:4649:2073 length=33
ATGACTATGACTACGACTACGACAAAGAAAAGA
>SRR1557864.73 HWI-D00318:201:H7P3BADXX:1:1101:4604:2081 length=33
TACCTGCATGGAGCCCTGTGCGAGTCGCTGAGG
>SRR1557864.74 HWI-D00318:201:H7P3BADXX:1:1101:4702:2155 length=33
GAAAGATTTGTCGACGGCATTTCCTAGTTTCGA
>SRR1557864.75 HWI-D00318:201:H7P3BADXX:1:1101:4586:2182 length=33
CGGCGCGATCCCGCCGCTGGTTGCGTTGCTCAT
>SRR1557864.76 HWI-D00318:201:H7P3BADXX:1:1101:4631:2186 length=33
GGATCTGGAGATCACAACAAGCAGCCCGAGCAC
>SRR1557864.77 HWI-D00318:201:H7P3BADXX:1:1101:4743:2203 length=33
TCAAACGAGGAAAGGCTTACGGTGGATACCTAG
>SRR1557864.78 HWI-D00318:201:H7P3BADXX:1:1101:4514:2204 length=33
CTAAGTGGCGGTGAGAAGGCTCGTCTCGCCTTC
>SRR1557864.79 HWI-D00318:201:H7P3BADXX:1:1101:4988:2039 length=33
GTGGCCAATTGGATCCGAAAAAGGTAGCTCAAG
>SRR1557864.80 HWI-D00318:201:H7P3BADXX:1:1101:4920:2040 length=33
AAGAGATTGTCATTGATCAAAGCATGGGAAGAA
>SRR1557864.81 HWI-D00318:201:H7P3BADXX:1:1101:5145:2060 length=33
GCCAACTCACAACATGTCTCCGGGGTCTCACGT
>SRR1557864.82 HWI-D00318:201:H7P3BADXX:1:1101:5146:2085 length=33
ATGGCATGATTCGGGTTTGGAAAGCCTGAAAAG
>SRR1557864.83 HWI-D00318:201:H7P3BADXX:1:1101:5096:2099 length=33
GGCGAGTCGGGTTGTTTGGGAATGCAGCCCCAA
>SRR1557864.84 HWI-D00318:201:H7P3BADXX:1:1101:5350:2076 length=33
CGGTTGAGAAGTACGACGACGTCGTACCCACTC
>SRR1557864.85 HWI-D00318:201:H7P3BADXX:1:1101:5427:2112 length=33
AAGCAGACCGGCCTCACCAGGAGTCAGGTATCA
>SRR1557864.86 HWI-D00318:201:H7P3BADXX:1:1101:5545:2115 length=33
ATTCAGCGGCGGACACCAAATACAAGGGCGTCC
>SRR1557864.87 HWI-D00318:201:H7P3BADXX:1:1101:5960:2054 length=33
GCAGCGACGCCGTGATCTATCCGGCGCCGGCGC
>SRR1557864.88 HWI-D00318:201:H7P3BADXX:1:1101:5895:2116 length=33
ATAAAGAAGGGTGTTTGCCCTATCATCGGGTTG

>SRR1557864.89 HWI-D00318:201:H7P3BADXX:1:1101:5925:2162 length=33
GTCAAGGTCTGTGAAGAGGAGAAGAAGGAAGAG
>SRR1557864.90 HWI-D00318:201:H7P3BADXX:1:1101:5820:2213 length=33
CCAATCACCTTGTCCTAGCCACAATGTCCGTGC
>SRR1557864.91 HWI-D00318:201:H7P3BADXX:1:1101:6103:2054 length=33
TTTTAAGTGAGGAAATTGTTGCTCCTTTTGGGG
>SRR1557864.92 HWI-D00318:201:H7P3BADXX:1:1101:6154:2083 length=33
TTCTGAGGAGGATGAACAGGTATGGTCTGCTGG
>SRR1557864.93 HWI-D00318:201:H7P3BADXX:1:1101:6199:2173 length=33
CTGCTTGGACTGCATGGGCAATGCCTTGTGCTC
>SRR1557864.94 HWI-D00318:201:H7P3BADXX:1:1101:6144:2178 length=33
GAAGAAGAAGAAAGAGAAGAAATCTGGAGAAGA
>SRR1557864.95 HWI-D00318:201:H7P3BADXX:1:1101:6396:2097 length=33
TTTAGTCCTAGCGAGGCTCTTGAATATGGAATT
>SRR1557864.96 HWI-D00318:201:H7P3BADXX:1:1101:6379:2143 length=33
GGGAAATGTAGGATTGTGTGGATTTCCTTTGAC
>SRR1557864.97 HWI-D00318:201:H7P3BADXX:1:1101:6454:2144 length=33
GCACTCAAACTCGCACAAGAAGGTCATTGAGAT
>SRR1557864.98 HWI-D00318:201:H7P3BADXX:1:1101:6265:2192 length=33
TCAAACGAGGAAAGGCTTACGGTGGATACCTAG
>SRR1557864.99 HWI-D00318:201:H7P3BADXX:1:1101:6284:2233 length=33
CTTCGAGGGGTTTAAGGTTTTGGCCAAGAACTA
>SRR1557864.100 HWI-D00318:201:H7P3BADXX:1:1101:6433:2246 length=33
GCTCATTTGGTAGCCAACAAGTGGCCCACTGGC
>SRR1557864.101 HWI-D00318:201:H7P3BADXX:1:1101:6395:2248 length=33
CTTAGATTGATTGTAAGTCGATGAAAGCAATTG
>SRR1557864.102 HWI-D00318:201:H7P3BADXX:1:1101:6620:2040 length=33
GCAGTAGATGAGCTAGCAGATGATGTAGAGAGA
>SRR1557864.103 HWI-D00318:201:H7P3BADXX:1:1101:6516:2068 length=33
TACCGCAGCACCACCGTGGAGTTCTTCTACAGG
>SRR1557864.104 HWI-D00318:201:H7P3BADXX:1:1101:6563:2106 length=33
AAAGAAAAGGGAAAACCAGCAAACGGGTACATT
>SRR1557864.105 HWI-D00318:201:H7P3BADXX:1:1101:6526:2164 length=33
GTGTTTTGGGGCTGTTGATTCTTGTTATTTATA
>SRR1557864.106 HWI-D00318:201:H7P3BADXX:1:1101:6953:2041 length=33
AAGATGAAGGAGCTTGAGGGCATCTGCAATCCG
>SRR1557864.107 HWI-D00318:201:H7P3BADXX:1:1101:6812:2107 length=33
AGCAGTGCGTTCGTGCTGAAACGTGCTAAAAGG
>SRR1557864.108 HWI-D00318:201:H7P3BADXX:1:1101:6793:2123 length=33
TATTGAATCAAAATGATGTTATTGACATCGCCG
>SRR1557864.109 HWI-D00318:201:H7P3BADXX:1:1101:6825:2127 length=33
CGATGCCGACGTCGCCGGCAGAAGCTTTCACAA
>SRR1557864.110 HWI-D00318:201:H7P3BADXX:1:1101:6827:2178 length=33
CTCTAGGCGCTCTTCTTGTGTATGATGTCACAA

>SRR1557864.111 HWI-D00318:201:H7P3BADXX:1:1101:6912:2190 length=33
ATCGGGAGCTTGGAGAGCAACGCGGGGCTGTTC
>SRR1557864.112 HWI-D00318:201:H7P3BADXX:1:1101:7197:2088 length=33
AGGACAAGGCGGGCGATGCAGGGAAGAAGGCGG
>SRR1557864.113 HWI-D00318:201:H7P3BADXX:1:1101:7394:2161 length=33
GTGTTTGGAGGATTTCGTGAAGATAGTGGAGGG
>SRR1557864.114 HWI-D00318:201:H7P3BADXX:1:1101:7512:2077 length=33
ACATGCACATTTACACACATAACACAGACTAAA
>SRR1557864.115 HWI-D00318:201:H7P3BADXX:1:1101:7633:2102 length=33
TGAGGGTTCGGACTCGAGCGACGAGGACGGAGC
>SRR1557864.116 HWI-D00318:201:H7P3BADXX:1:1101:7744:2190 length=33
TGAGTTGTGGCTCATTTTCTTGTGTCGTTAGTC
>SRR1557864.117 HWI-D00318:201:H7P3BADXX:1:1101:7735:2219 length=33
ATCCGCTCGATCATGCCGCCTACCATCTCGCGA
>SRR1557864.118 HWI-D00318:201:H7P3BADXX:1:1101:7750:2042 length=33
GAAGATTCAGCGGCGGACACCAAATACAAGGGC
>SRR1557864.119 HWI-D00318:201:H7P3BADXX:1:1101:7847:2046 length=33
GCCAAGATCCAGGACAAGGAGGGTATTCCCCCG
>SRR1557864.120 HWI-D00318:201:H7P3BADXX:1:1101:7982:2082 length=33
GCATGGGAAGCTATGGGTATTCGAGTTTTGGGA
>SRR1557864.121 HWI-D00318:201:H7P3BADXX:1:1101:7757:2101 length=33
TAGTCGGGCTGGCGACTTAAAAACTAGCGCTGC
>SRR1557864.122 HWI-D00318:201:H7P3BADXX:1:1101:7957:2122 length=33
ACAAGCCTGTCGTAGTGAAGGCGAAGCTGGTGT
>SRR1557864.123 HWI-D00318:201:H7P3BADXX:1:1101:7936:2149 length=33
TCAAACGAGGAAAGGCTTACGGTGGATACCTAG
>SRR1557864.124 HWI-D00318:201:H7P3BADXX:1:1101:7989:2248 length=33
AGCATATCAATAAGCGGAGGAAAAGAAACTTAC
>SRR1557864.125 HWI-D00318:201:H7P3BADXX:1:1101:8176:2045 length=33
AGGCGGAGTACTGGTCGATGGTGTCGAGCTTCT
>SRR1557864.126 HWI-D00318:201:H7P3BADXX:1:1101:8226:2139 length=33
GAGGCTGATGAGTTTGAGGACAAGATGAAGGAG
>SRR1557864.127 HWI-D00318:201:H7P3BADXX:1:1101:8173:2176 length=33
GTAGGAAACAAACGACCGCCCAAAACAAGGCAA
>SRR1557864.128 HWI-D00318:201:H7P3BADXX:1:1101:8143:2177 length=33
CACAGAATGTTGAAGCTTGGTTTGAGCATTGAC
>SRR1557864.129 HWI-D00318:201:H7P3BADXX:1:1101:8236:2191 length=33
TGGAGAAGTCGGCTCTGTGGCAGCGCGCCAGCA
>SRR1557864.130 HWI-D00318:201:H7P3BADXX:1:1101:8050:2209 length=33
TTCAAAGGGAATGCGGGGTTGTGTGGTTTCAAT
>SRR1557864.131 HWI-D00318:201:H7P3BADXX:1:1101:8325:2037 length=33
AACGACGGACGCGCCAAGGCGGCCAACGTGACC
>SRR1557864.132 HWI-D00318:201:H7P3BADXX:1:1101:8474:2051 length=33
TGAAGAAGATGGGTGAGGGGAAGGACGCGCACA

>SRR1557864.133 HWI-D00318:201:H7P3BADXX:1:1101:8381:2056 length=33
GTCTGGAAATGTGTGCTGAGAGAGCTGTCGCGT
>SRR1557864.134 HWI-D00318:201:H7P3BADXX:1:1101:8415:2093 length=33
ATCCCTCAACCAAAAAGACTGAGTGGACCCGAG
>SRR1557864.135 HWI-D00318:201:H7P3BADXX:1:1101:8316:2112 length=33
CCATGTTCAGGCGCAAGGCCTTCTTGCATTGGT
>SRR1557864.136 HWI-D00318:201:H7P3BADXX:1:1101:8314:2165 length=33
CGAGGAATTGTTACGAGAGGGCCGTGAATAAGT
>SRR1557864.137 HWI-D00318:201:H7P3BADXX:1:1101:8427:2211 length=33
TGTCGGATGCAATTTCAGCAGAGTTTGCATTTG
>SRR1557864.138 HWI-D00318:201:H7P3BADXX:1:1101:8633:2047 length=33
GAGGAAGAAGTTGGAGAAGACGGGTTGAAGAAG
>SRR1557864.139 HWI-D00318:201:H7P3BADXX:1:1101:8609:2065 length=33
AGAATGCATTGGATGGATGCCCGGGCATTGAGA
>SRR1557864.140 HWI-D00318:201:H7P3BADXX:1:1101:8712:2091 length=33
TCAAACGAGGAAAGGCTTACGGTGGATACCTAG
>SRR1557864.141 HWI-D00318:201:H7P3BADXX:1:1101:8653:2109 length=33
CGAAGGCGACGACAGCAAGAAGAAGAAGAAGGA
>SRR1557864.142 HWI-D00318:201:H7P3BADXX:1:1101:8515:
>SRR1557864.658361 HWI-D00318:201:H7P3BADXX:1:1104:10060:44506 length=33
CTGCACATGGTTGGACATTACCTACTCAGGAAG
>SRR1557864.658362 HWI-D00318:201:H7P3BADXX:1:1104:10121:44512 length=33
ACAGGGGGAATAAGGAGAGCGGTAAGGATAGCA
>SRR1557864.658363 HWI-D00318:201:H7P3BADXX:1:1104:10148:44521 length=33
TCAAACGAGGAAAGGCTTACGGTGGATACCTAG
>SRR1557864.658364 HWI-D00318:201:H7P3BADXX:1:1104:10207:44557 length=33
TCAAACGAGGAAAGGCTTACGGTGGATACCTAG
>SRR1557864.658365 HWI-D00318:201:H7P3BADXX:1:1104:10189:44581 length=33
GATCAAATCCTTGATATCAATATTAGCAAGACA
>SRR1557864.658366 HWI-D00318:201:H7P3BADXX:1:1104:10050:44628 length=33
GATTGTGAGCCCAAGGTGCAGCCCTCGATTCAT
>SRR1557864.658367 HWI-D00318:201:H7P3BADXX:1:1104:10138:44662 length=33
TCGCTCTAGTGACCTCTCATCACGGATGTAATG
>SRR1557864.658368 HWI-D00318:201:H7P3BADXX:1:1104:10036:44689 length=33
GCGACCCCAGGTCAGGCGGGATTACCCGCTGAG
>SRR1557864.658369 HWI-D00318:201:H7P3BADXX:1:1104:10116:44734 length=33
CAATAGTTATGCATATAGTATTATATATGTTTG
>SRR1557864.658370 HWI-D00318:201:H7P3BADXX:1:1104:10163:44741 length=33
ACCCTACCCTATCCGTATTCAACGACGTCATTA
>SRR1557864.658371 HWI-D00318:201:H7P3BADXX:1:1104:10354:44545 length=33
CAGTGCACTTTCGTCGAATCGCCGCTCTACGCC
>SRR1557864.658372 HWI-D00318:201:H7P3BADXX:1:1104:10392:44578 length=33
AGTACCAGGATGCGACGGCCGACGAGGAGGGTG
>SRR1557864.658373 HWI-D00318:201:H7P3BADXX:1:1104:10374:44602 length=33

CCCCAACGATGTCGAGTGGGAGCAGGAAGAGTC
>SRR1557864.658374 HWI-D00318:201:H7P3BADXX:1:1104:10452:44612 length=33
TCAAACGAGGAAAGGCTTACGGTGGATACCTAG
>SRR1557864.658375 HWI-D00318:201:H7P3BADXX:1:1104:10465:44625 length=33
TCAAACGAGGAAAGGCTTACGGTGGATACCTAG
>SRR1557864.658376 HWI-D00318:201:H7P3BADXX:1:1104:10309:44656 length=33
GACGCTGTGGTTCTGTTCTTGTGCGCCTGATTC
>SRR1557864.658377 HWI-D00318:201:H7P3BADXX:1:1104:10348:44667 length=33
AGAGGAAGGCGAGGGTAAGGCGAATAAGAAGAG
>SRR1557864.658378 HWI-D00318:201:H7P3BADXX:1:1104:10323:44672 length=33
GGGTGTTTTGAAATTGTGAAAATGGAATTTGAG
>SRR1557864.658379 HWI-D00318:201:H7P3BADXX:1:1104:10474:44698 length=33
TCAAACGAGGAAAGGCTTACGATGGATACCTAG
>SRR1557864.658380 HWI-D00318:201:H7P3BADXX:1:1104:10354:44735 length=33
GGGAATAAGCATCGGCTAACTCTGTGCCAGCAG
>SRR1557864.658381 HWI-D00318:201:H7P3BADXX:1:1104:10326:44740 length=33
GCCGCCCGCCGCCCCAGATCTCCAATCTCTTCG
>SRR1557864.658382 HWI-D00318:201:H7P3BADXX:1:1104:10498:44745 length=33
CTACCAAAATTCACTTTCCTCGCGAGGATGATC
>SRR1557864.658383 HWI-D00318:201:H7P3BADXX:1:1104:10703:44515 length=33
GTACCCACTCAAGAGCATGAGGAGAAGAAAGGC
>SRR1557864.658384 HWI-D00318:201:H7P3BADXX:1:1104:10645:44545 length=33
CTGACTGATTTGTTATTATTATTTTTCCCTTTT
>SRR1557864.658385 HWI-D00318:201:H7P3BADXX:1:1104:10625:44580 length=33
AGATGAGCTGTTTGAAAGACTTGAGTACCGTTC
>SRR1557864.658386 HWI-D00318:201:H7P3BADXX:1:1104:10662:44647 length=33
GGCGAGATATTCCAGATTTTTGAGGAGAGAGAG
>SRR1557864.658387 HWI-D00318:201:H7P3BADXX:1:1104:10679:44664 length=33
CGGGAAGGCGTACTACGGAGGGAACGTGGAGAA
>SRR1557864.658388 HWI-D00318:201:H7P3BADXX:1:1104:10617:44672 length=33
GTATATAAAAGCTTTCTATGAGAAGACAAAGCT
>SRR1557864.658389 HWI-D00318:201:H7P3BADXX:1:1104:10645:44685 length=33
AACGTGGTGACCGCCCAAATCAAGTACACGGCA
>SRR1557864.658390 HWI-D00318:201:H7P3BADXX:1:1104:10506:44717 length=33
AGAATGCATTGGATGGATGCCCGGGCATTGAGA
>SRR1557864.658391 HWI-D00318:201:H7P3BADXX:1:1104:10725:44728 length=33
CGAGTCGGGTTGTTTGGGAATGCAGCCCCAATC
>SRR1557864.658392 HWI-D00318:201:H7P3BADXX:1:1104:10994:44562 length=33
AGAATGCATTGGATGGATGCCCGGGCATTGAGA
>SRR1557864.658393 HWI-D00318:201:H7P3BADXX:1:1104:10922:44580 length=33
ACGATCCGGAGTACGCGGTCTTCCTCTCTGACC
>SRR1557864.658394 HWI-D00318:201:H7P3BADXX:1:1104:10953:44619 length=33
AAGGGAGAAACAAATACAAAAACTGCTTCTTAG
>SRR1557864.658395 HWI-D00318:201:H7P3BADXX:1:1104:10878:44675 length=33

GGTTTGGTAGTTCAGACAGTTCTATACCTGGTG
>SRR1557864.658396 HWI-D00318:201:H7P3BADXX:1:1104:11166:44572 length=33
GGAGAGTCTGAAAGGTCCGAATATGTGTCAATA
>SRR1557864.658397 HWI-D00318:201:H7P3BADXX:1:1104:11203:44596 length=33
CGGGCAGCCGGTCATCCTCTTCGATCCGGCCAT
>SRR1557864.658398 HWI-D00318:201:H7P3BADXX:1:1104:11026:44628 length=33
ATTTAGGCTCGTTTAATGCTGGGGATCACGCAA
>SRR1557864.658399 HWI-D00318:201:H7P3BADXX:1:1104:11203:44678 length=33
CAGGAAAGAGAAGAGACGATCAGCAACATTATC
>SRR1557864.658400 HWI-D00318:201:H7P3BADXX:1:1104:11036:44714 length=33
TCTCATGGAGAGTTCGATCCTGGCTCAGGATGA
>SRR1557864.658401 HWI-D00318:201:H7P3BADXX:1:1104:11161:44735 length=33
GGAATGTTGGGGCGAACCAGAAAAGTTTGGGTA
>SRR1557864.658402 HWI-D00318:201:H7P3BADXX:1:1104:11291:44552 length=33
ACGACATGAAGTTCGAGGACACGGAAGATGGCG
>SRR1557864.658403 HWI-D00318:201:H7P3BADXX:1:1104:11255:44597 length=33
GACCTTGGATATGATGCAGCTAAAGGTGAATAT
>SRR1557864.658404 HWI-D00318:201:H7P3BADXX:1:1104:11448:44603 length=33
CCCTTCAGCTTTGAGCAAATGCTGGAGACAGGT
>SRR1557864.658405 HWI-D00318:201:H7P3BADXX:1:1104:11256:44638 length=33
GAAGAAAGGGTTACTAGACAAGATCAAGGAGAA
>SRR1557864.658406 HWI-D00318:201:H7P3BADXX:1:1104:11380:44717 length=33
GAGGAAGGTCCTCTTGAAAGCTTTCGGAGCTAA
>SRR1557864.658407 HWI-D00318:201:H7P3BADXX:1:1104:11270:44726 length=33
ATCCAGCACTTGTTCTCAGTATTCAGGCTTATG
>SRR1557864.658408 HWI-D00318:201:H7P3BADXX:1:1104:11372:44736 length=33
CTCAGGAATCCCACCTGCACCGAGAGGAGTTCC
>SRR1557864.658409 HWI-D00318:201:H7P3BADXX:1:1104:11684:44518 length=33
AGATGAAATGTTGAGGTTTTGTGGGTTTTTGGA
>SRR1557864.658410 HWI-D00318:201:H7P3BADXX:1:1104:11640:44602 length=33
ATTTGATAAAATTTATTGAAGCTAGCGTTGCGA
>SRR1557864.658411 HWI-D00318:201:H7P3BADXX:1:1104:11680:44606 length=33
ACGGATCTGGAGATCTCCACAAGCAGCCCGAAC
>SRR1557864.658412 HWI-D00318:201:H7P3BADXX:1:1104:11646:44624 length=33
CGGCCACCATCGTGTCTCAGTACAGCGGAAATC
>SRR1557864.658413 HWI-D00318:201:H7P3BADXX:1:1104:11722:44726 length=33
CAGGCTCTGGCTTCGGACAAAAGGAAGGGGTTT
>SRR1557864.658414 HWI-D00318:201:H7P3BADXX:1:1104:11997:44511 length=33
TAAGATTGTTCCACCGGCGAGTTGGAAACCCCC
>SRR1557864.658415 HWI-D00318:201:H7P3BADXX:1:1104:11896:44559 length=33
ATATCTGCGGATGACAACGGTGCATTAGCCGGA
>SRR1557864.658416 HWI-D00318:201:H7P3BADXX:1:1104:11970:44572 length=33
GTTCAGAAATAGTTAGGAAATCCATGATTACAC
>SRR1557864.658417 HWI-D00318:201:H7P3BADXX:1:1104:11978:44586 length=33

GTCACAACTGCTGGGGAATTATCAAAAAAGCCG
>SRR1557864.658418 HWI-D00318:201:H7P3BADXX:1:1104:11859:44613 length=33
GTACGTAAGAAGGAGGTGGAAGAGTCACGTAAG
>SRR1557864.658419 HWI-D00318:201:H7P3BADXX:1:1104:11953:44688 length=33
CGGCGGCCGCGATTTCCAGCTGAGTTCGAAGGA
>SRR1557864.658420 HWI-D00318:201:H7P3BADXX:1:1104:11955:44743 length=33
TGGAGTAGTAAAAGCAATTGGAACTCAGGGGGT
>SRR1557864.658421 HWI-D00318:201:H7P3BADXX:1:1104:11830:44743 length=33
CCTGCAATGCTTTACTACTGAAGGTGAACCAAA
>SRR1557864.658422 HWI-D00318:201:H7P3BADXX:1:1104:11986:44748 length=33
AGTCTGGAGTGTGAATTCATTACAAATAAGCAT
>SRR1557864.658423 HWI-D00318:201:H7P3BADXX:1:1104:12080:44507 length=33
GGCCTTGCTGAACTTCCCGCACAGGATCGGCTT
>SRR1557864.658424 HWI-D00318:201:H7P3BADXX:1:1104:12207:44527 length=33
CCTATACCTTCTCATTTGATTATCCATCTCAAA
>SRR1557864.658425 HWI-D00318:201:H7P3BADXX:1:1104:12164:44536 length=33
GAGCCTTTCGTGGAAAAGCTACACCTCATGCGG
>SRR1557864.658426 HWI-D00318:201:H7P3BADXX:1:1104:12020:44557 length=33
CGATCGGCGGCGAGATGGCGGCGGCGGAGGCGG
>SRR1557864.658427 HWI-D00318:201:H7P3BADXX:1:1104:12058:44558 length=33
CCCGCAGTATCAGAAATCCACTGCGGCGTACCG
>SRR1557864.658428 HWI-D00318:201:H7P3BADXX:1:1104:12228:44597 length=33
GACTGAGTAGTGCGTGTCGATGAGGTGGACGAT
>SRR1557864.658429 HWI-D00318:201:H7P3BADXX:1:1104:12002:44640 length=33
TCTGAAGATGCAGCTGGAGCTGTTAAGCAACTT
>SRR1557864.658430 HWI-D00318:201:H7P3BADXX:1:1104:12209:44647 length=33
GCTGAAGCTGAGGGCAGCAAGATGGAGGAAGTC
>SRR1557864.658431 HWI-D00318:201:H7P3BADXX:1:1104:12229:44677 length=33
ACGGCGTCGAAGGCGCCGATTCCTTCAGCTTCA
>SRR1557864.658432 HWI-D00318:201:H7P3BADXX:1:1104:12434:44542 length=33
GTGCCAATTGTTGAAGAAGAAGTCCCGGTGGCT
>SRR1557864.658433 HWI-D00318:201:H7P3BADXX:1:1104:12287:44585 length=33
AAATATGATGATGCTGACCTCCCCTGTGAAGCT
>SRR1557864.658434 HWI-D00318:201:H7P3BADXX:1:1104:12262:44691 length=33
CTATTGCTATTGGCCCTTGTGTCCGTCCCGTGG
>SRR1557864.658435 HWI-D00318:201:H7P3BADXX:1:1104:12473:44692 length=33
CATGACCGTCTTCATCTTCTTCTTCCTGCCGGA
>SRR1557864.658436 HWI-D00318:201:H7P3BADXX:1:1104:12484:44716 length=33
AGAATGCATTGGATGGATGCCCGGGCATTGAGA
>SRR1557864.658437 HWI-D00318:201:H7P3BADXX:1:1104:12464:44731 length=33
GGCGCCAAGAAGGAGGAGGAGACGGCGATCTCC
>SRR1557864.658438 HWI-D00318:201:H7P3BADXX:1:1104:12569:44547 length=33
GTGATGAGGCTGTGAAAGAGGATGATGATATGT
>SRR1557864.658439 HWI-D00318:201:H7P3BADXX:1:1104:12625:44558 length=33

AGAGGAAGCTGAATGAAGTCGCTGATCCAAGGC
>SRR1557864.658440 HWI-D00318:201:H7P3BADXX:1:1104:12518:44600 length=33
AGAGGTTAAGTGCGTAGACACATCGGTTCCGGT
>SRR1557864.658441 HWI-D00318:201:H7P3BADXX:1:1104:12697:44655 length=33
AGAATGCATTGGATGGATGCCCGGGCATTGAGA
>SRR1557864.658442 HWI-D00318:201:H7P3BADXX:1:1104:12720:44685 length=33
CTTTAGGTCCAAGGCCAAGTCCTCTGAATGAAT
>SRR1557864.658443 HWI-D00318:201:H7P3BADXX:1:1104:12569:44695 length=33
TCAAAGAGAAAGTTGCTTTCAAGACTAAATCAG
>SRR1557864.658444 HWI-D00318:201:H7P3BADXX:1:1104:12671:44740 length=33
TCAAACGAGGAAAGGCTTACGGTGGATACCTAG
>SRR1557864.658445 HWI-D00318:201:H7P3BADXX:1:1104:12786:44504 length=33
GCCGTGATCACGACGTACGAGGGGAAGCACAAC
>SRR1557864.658446 HWI-D00318:201:H7P3BADXX:1:1104:12812:44534 length=33
TGGCGCAGTACAATTCGCCGTTTGAGTTCAGGA
>SRR1557864.658447 HWI-D00318:201:H7P3BADXX:1:1104:12959:44550 length=33
GTAGGCAAGGGAAGTCGGCAAAATGGATCCGTA
>SRR1557864.658448 HWI-D00318:201:H7P3BADXX:1:1104:12802:44551 length=33
TTCATCGTGAACGTGATCAAAACCGGGGTGAGA
>SRR1557864.658449 HWI-D00318:201:H7P3BADXX:1:1104:12997:44577 length=33
CGGAATGGAGCGAGAGGCAGCGAAGGAGGAAGC
>SRR1557864.658450 HWI-D00318:201:H7P3BADXX:1:1104:12876:44634 length=33
GAAGGACCCTGCGGCGGCGCTCTACAGAGTGGC
>SRR1557864.658451 HWI-D00318:201:H7P3BADXX:1:1104:12765:44636 length=33
GTGTGGAGAAAGAAGATTGAGAAGGATGTGTCG
>SRR1557864.658452 HWI-D00318:201:H7P3BADXX:1:1104:12754:44729 length=33
TCAAACGAGGAAAGGCTTACGGTGGATACCTAG
>SRR1557864.658453 HWI-D00318:201:H7P3BADXX:1:1104:12987:44729 length=33
CTTTTACTCTATTCTTGTTTTTCTTCCCTCTTC
>SRR1557864.658454 HWI-D00318:201:H7P3BADXX:1:1104:13199:44722 length=33
CTGCTATTTTTACAATTATTTTATTCTACTAAA
>SRR1557864.658455 HWI-D00318:201:H7P3BADXX:1:1104:13344:44564 length=33
GGATGGGTTTCGTAATGTGCTGATGGTCTGTGC
>SRR1557864.658456 HWI-D00318:201:H7P3BADXX:1:1104:13428:44631 length=33
AAGCCGATGAGGAAGAATGCGAAGATGATGAAG
>SRR1557864.658457 HWI-D00318:201:H7P3BADXX:1:1104:13410:44644 length=33
CGGGATTACCCGCTGAGTTTAAGCATATCAATA
>SRR1557864.658458 HWI-D00318:201:H7P3BADXX:1:1104:13446:44657 length=33
TCAGAGGCCGAAGCTCTAGTTTCGAGTGAGAAG
>SRR1557864.658459 HWI-D00318:201:H7P3BADXX:1:1104:13479:44672 length=33
TCAAACGAGGAAAGGCTTACGGTGGATACCTAG
>SRR1557864.658460 HWI-D00318:201:H7P3BADXX:1:1104:13497:44676 length=33
GATCGCGCAGACCGACGGCACGCGGATGTCGCT
>SRR1557864.658461 HWI-D00318:201:H7P3BADXX:1:1104:13588:44550 length=33

TCAAACGAGGAAAGGCTTACGGTGGATACCTAG
>SRR1557864.658462 HWI-D00318:201:H7P3BADXX:1:1104:13639:44571 length=33
AAGTTTGCAAAGAGATAGAGGCGTCTGGTGGAC
>SRR1557864.658463 HWI-D00318:201:H7P3BADXX:1:1104:13533:44577 length=33
GATTCAGGATAAGGAAGGGATCCCACCGGACCA
>SRR1557864.658464 HWI-D00318:201:H7P3BADXX:1:1104:13534:44644 length=33
CAAGCATCAGAAGAAATGTTCTACCTGTGGATC
>SRR1557864.658465 HWI-D00318:201:H7P3BADXX:1:1104:13826:44520 length=33
TCAAACGAGGAAAGGCTTACGGTGGATACCTAG
>SRR1557864.658466 HWI-D00318:201:H7P3BADXX:1:1104:13892:44561 length=33
CCACGCCGCCCGGGCTGACGGAACCCCCGCCAA
>SRR1557864.658467 HWI-D00318:201:H7P3BADXX:1:1104:13862:44574 length=33
AGCTACCCGTTGGAGGTGGCGAGAAAGCGGCTA
>SRR1557864.658468 HWI-D00318:201:H7P3BADXX:1:1104:13894:44604 length=33
CAAAGAAGAGTTGGCAGCGGAAAAGCTGAGGAC
>SRR1557864.658469 HWI-D00318:201:H7P3BADXX:1:1104:13936:44618 length=33
CCGACTCCCCCAATTTCAGTGTAGCAACGCCGG
>SRR1557864.658470 HWI-D00318:201:H7P3BADXX:1:1104:14051:44530 length=33
AACAAGAGAAGAAAACAAATTCCACTAATTTCT
>SRR1557864.658471 HWI-D00318:201:H7P3BADXX:1:1104:14012:44547 length=33
GAGAGAGAGAGAGGTGGGGTGAATATGTGAAAG
>SRR1557864.658472 HWI-D00318:201:H7P3BADXX:1:1104:14163:44556 length=33
CCTCTGTGGCGGATACATCCAGCAGCAGCGGGC
>SRR1557864.658473 HWI-D00318:201:H7P3BADXX:1:1104:14049:44556 length=33
ACACGCAGCGCCACAGGCTTGGGCCCAACCACC
>SRR1557864.658474 HWI-D00318:201:H7P3BADXX:1:1104:14034:44559 length=33
AGAAGAAGAAGAAAAAGAAGGGTTTGAAGGAGA
>SRR1557864.658475 HWI-D00318:201:H7P3BADXX:1:1104:14194:44618 length=33
GTGGCCGTGGAGGTGACGCGCCTGGCCCCACAC
>SRR1557864.658476 HWI-D00318:201:H7P3BADXX:1:1104:14101:44676 length=33
AGCCATCCGATCACCACGGTCTTGCACGTTGAA
>SRR1557864.658477 HWI-D00318:201:H7P3BADXX:1:1104:14123:44748 length=33
GAACGTGGTGACCGCCCAAATCAAGTACACGGC
>SRR1557864.658478 HWI-D00318:201:H7P3BADXX:1:1104:14316:44534 length=33
GGTCGAACTATGGGTAACAAGCAGTCGAGGATG
>SRR1557864.658479 HWI-D00318:201:H7P3BADXX:1:1104:14471:44570 length=33
GTGAAGAAGGTCATCTGGCTGCCTCATGGATTG
>SRR1557864.658480 HWI-D00318:201:H7P3BADXX:1:1104:14497:44606 length=33
AGAGGTGAATGGCACTGTTCTGGCTAGTGGAGG
>SRR1557864.658481 HWI-D00318:201:H7P3BADXX:1:1104:14305:44643 length=33
GGACGGAGACGGGCTGCTGTGTTTGGAGGATTT
>SRR1557864.658482 HWI-D00318:201:H7P3BADXX:1:1104:14349:44662 length=33
GCAACCTCGTTGAGATCCAAGGTTGGATTAGGT
>SRR1557864.658483 HWI-D00318:201:H7P3BADXX:1:1104:14305:44680 length=33

GCGGGAGAGCCGGCGAAGCTGATCAGGCAGCGC
>SRR1557864.658484 HWI-D00318:201:H7P3BADXX:1:1104:14459:44739 length=33
ATTCTGCCCAAAAATCCTTACGACCGAGCCATG
>SRR1557864.658485 HWI-D00318:201:H7P3BADXX:1:1104:14654:44567 length=33
GGGCAGGCCTTGGGAGGACACAAGCGCCGCCAC
>SRR1557864.658486 HWI-D00318:201:H7P3BADXX:1:1104:14597:44579 length=33
CCGATTTCCGATCTAAAATCTCAGAATTCATCC
>SRR1557864.658487 HWI-D00318:201:H7P3BADXX:1:1104:14683:44651 length=33
TTGCTGCTGCAGATAATGTGAGCGGTGTTGTCG
>SRR1557864.658488 HWI-D00318:201:H7P3BADXX:1:1104:14638:44669 length=33
GGTTATGCCTGGCTGGCAGGTCGATATTTCTTC
>SRR1557864.658489 HWI-D00318:201:H7P3BADXX:1:1104:14697:44677 length=33
ATGAGTTGGGTGTATCACAAAGCCAAGTGCAGC
>SRR1557864.658490 HWI-D00318:201:H7P3BADXX:1:1104:14552:44725 length=33
TTTTACGTTGAACATTATTGACACCCCTGGACT
>SRR1557864.658491 HWI-D00318:201:H7P3BADXX:1:1104:14938:44518 length=33
ACTTTGAAAAGAGAGTCAAAGAGTGCTTGAAAT
>SRR1557864.658492 HWI-D00318:201:H7P3BADXX:1:1104:14814:44593 length=33
GATGTCGCTCGCCTCCTTCTACAACCCCGGCAG
>SRR1557864.658493 HWI-D00318:201:H7P3BADXX:1:1104:14876:44678 length=33
ACGCCTAGAAGATGGATCGGTTTCTGCAATCCG
>SRR1557864.658494 HWI-D00318:201:H7P3BADXX:1:1104:14898:44740 length=33
AACTGAGACAAAAACATGCCCTTCCATCTGGAG
>SRR1557864.658495 HWI-D00318:201:H7P3BADXX:1:1104:15027:44530 length=33
TCAAACGAGGAAAGGCTTACGGTGGATACCTAG
>SRR1557864.658496 HWI-D00318:201:H7P3BADXX:1:1104:15194:44635 length=33
GAACATGGCTACGGATCTGGAGATCACAACAAG
>SRR1557864.658497 HWI-D00318:201:H7P3BADXX:1:1104:15182:44663 length=33
CACGCTGTTTTTCGACGACAATGTGAAGAATAT
>SRR1557864.658498 HWI-D00318:201:H7P3BADXX:1:1104:15131:44687 length=33
TTCTCGCAGACCTGCAACCTTTTGAGCCAGTAT
>SRR1557864.658499 HWI-D00318:201:H7P3BADXX:1:1104:15006:44720 length=33
TCAGACTCAGATCCAGCGTCTGCTCTGTCGCGC
>SRR1557864.658500 HWI-D00318:201:H7P3BADXX:1:1104:15065:44726 length=33
TCAAACGAGGAAAGGCTTACGGTGGATACCTAG
>SRR1557864.658501 HWI-D00318:201:H7P3BADXX:1:1104:15272:44527 length=33
CCCTACACGACGCTCTTCCGATCTCTCCCCTAC
>SRR1557864.658502 HWI-D00318:201:H7P3BADXX:1:1104:15414:44536 length=33
CCATGGTTCCAAACCTACGAAACATGCGGCAAC
>SRR1557864.658503 HWI-D00318:201:H7P3BADXX:1:1104:15461:44542 length=33
AGATGCTCCTCAATCTTCACAAAAAGAAATGGA
>SRR1557864.658504 HWI-D00318:201:H7P3BADXX:1:1104:15389:44577 length=33
CGTCGGACTCGGGCGTCCTCACCCCGAGGAGCG
>SRR1557864.658505 HWI-D00318:201:H7P3BADXX:1:1104:15259:44584 length=33

ATCTCTCTCGACATATATGGAGGATGATCGAAG
>SRR1557864.658506 HWI-D00318:201:H7P3BADXX:1:1104:15295:44598 length=33
TGAGGTTCTAAGGCAAAGGATATTGGATTTGGA
>SRR1557864.658507 HWI-D00318:201:H7P3BADXX:1:1104:15439:44664 length=33
CCTACACGACGCTCTTCCGATCTGCCCCTACAC
>SRR1557864.658508 HWI-D00318:201:H7P3BADXX:1:1104:15348:44731 length=33
TTCTCCCGAAGAAGACAGGGTCGTCGAAGCCAT
>SRR1557864.658509 HWI-D00318:201:H7P3BADXX:1:1104:15630:44530 length=33
CCAAACTGAGCTTTTCCATCGACAAAAGAAATG
>SRR1557864.658510 HWI-D00318:201:H7P3BADXX:1:1104:15525:44563 length=33
GTCGGCGAGTCGGGTTGTTTGGGAATGCAGCCC
>SRR1557864.658511 HWI-D00318:201:H7P3BADXX:1:1104:15677:44592 length=33
GCACTCAAACTCGCACAAGAAGGTCATTGAGAT
>SRR1557864.658512 HWI-D00318:201:H7P3BADXX:1:1104:15655:44673 length=33
GGAGGGAAGCGGATGGGGGCCGGCGATGTGTCC
>SRR1557864.658513 HWI-D00318:201:H7P3BADXX:1:1104:15705:44697 length=33
CACAACGAAGTGGAGCTTTCGGCTCTCGGAATG
>SRR1557864.658514 HWI-D00318:201:H7P3BADXX:1:1104:15700:44748 length=33
ATCAATCAGCGATGAAGAAGATGAGCCGGAGCG
>SRR1557864.658515 HWI-D00318:201:H7P3BADXX:1:1104:15936:44517 length=33
TGCAGCGGCGGCATCCGCCGCGACGTCGTTGAT
>SRR1557864.658516 HWI-D00318:201:H7P3BADXX:1:1104:15809:44546 length=33
AGCAATACGAGCGGGTTTTCAACCACTTCGATT
>SRR1557864.658517 HWI-D00318:201:H7P3BADXX:1:1104:15964:44551 length=33
GTATAAGACCGACCAGTACGGCGCCACCGGAAC
>SRR1557864.658518 HWI-D00318:201:H7P3BADXX:1:1104:15978:44608 length=33
CTCTTCGAGCTCGCAGCGGCAGAAGGAAGCGGC
>SRR1557864.658519 HWI-D00318:201:H7P3BADXX:1:1104:15808:44629 length=33
TCTTCCGGCTCTTGATCAGTTGCTGAAAGATCC
>SRR1557864.658520 HWI-D00318:201:H7P3BADXX:1:1104:15762:44645 length=33
ACCGACCAGTACGGCGCCACCGGAACCACCGGG
>SRR1557864.658521 HWI-D00318:201:H7P3BADXX:1:1104:16056:44543 length=33
CACCACAAGCAGCCGGCAACCGAGCACGAGGCA
>SRR1557864.658522 HWI-D00318:201:H7P3BADXX:1:1104:16029:44547 length=33
GCTGGTTACGGACAGTACCCCCATCAGACCGAA
>SRR1557864.658523 HWI-D00318:201:H7P3BADXX:1:1104:16050:44587 length=33
AGAATGCATTGGATGGATGCCCGGGCATTGAGA
>SRR1557864.658524 HWI-D00318:201:H7P3BADXX:1:1104:16204:44654 length=33
GAGAGAACAAGGACCAGGGATAACAACTTGCTT
>SRR1557864.658525 HWI-D00318:201:H7P3BADXX:1:1104:16146:44670 length=33
CTGCATCGTCTCCAACAGCACTAAAGCTCAGTG
>SRR1557864.658526 HWI-D00318:201:H7P3BADXX:1:1104:16241:44718 length=33
TATTCTGCACACTTCAATGAAATCTCCGAGCAG
>SRR1557864.658527 HWI-D00318:201:H7P3BADXX:1:1104:16110:44739 length=33

GAAAGTACGGTACCCGTTACGGTGCCTCGCTCC
>SRR1557864.658528 HWI-D00318:201:H7P3BADXX:1:1104:16407:44505 length=33
GTTAAGTCTGTTGAGATGCACCACGAGGCCCTG
>SRR1557864.658529 HWI<div id="preview-all"><div class="button" onclick="Sb('T',1)"
